# Age and Microenvironment Outweigh Genetic Influence on the Zucker Rat Microbiome

**DOI:** 10.1371/journal.pone.0100916

**Published:** 2014-09-18

**Authors:** Hannah Lees, Jonathan Swann, Simon M. Poucher, Jeremy K. Nicholson, Elaine Holmes, Ian D. Wilson, Julian R. Marchesi

**Affiliations:** 1 Section of Computational and Systems Medicine, Department of Surgery and Cancer, Faculty of Medicine, Imperial College London, London, United Kingdom; 2 Department of Food and Nutritional Sciences, School of Chemistry, Food and Pharmacy, University of Reading, Reading, United Kingdom; 3 Cardiovascular and Gastro-Intestinal Disorders Innovative Medicines, AstraZeneca Pharmaceuticals, Alderley Park, Cheshire, United Kingdom; 4 School of Biosciences, Cardiff University, Cardiff, United Kingdom; 5 Centre for Digestive and Gut Health, Imperial College London, London, United Kingdom; Instutite of Agrochemistry and Food Technology, Spain

## Abstract

Animal models are invaluable tools which allow us to investigate the microbiome-host dialogue. However, experimental design introduces biases in the data that we collect, also potentially leading to biased conclusions. With obesity at pandemic levels animal models of this disease have been developed; we investigated the role of experimental design on one such rodent model. We used 454 pyrosequencing to profile the faecal bacteria of obese (n = 6) and lean (homozygous n = 6; heterozygous n = 6) Zucker rats over a 10 week period, maintained in mixed-genotype cages, to further understand the relationships between the composition of the intestinal bacteria and age, obesity progression, genetic background and cage environment. Phylogenetic and taxon-based univariate and multivariate analyses (non-metric multidimensional scaling, principal component analysis) showed that age was the most significant source of variation in the composition of the faecal microbiota. Second to this, cage environment was found to clearly impact the composition of the faecal microbiota, with samples from animals from within the same cage showing high community structure concordance, but large differences seen between cages. Importantly, the genetically induced obese phenotype was not found to impact the faecal bacterial profiles. These findings demonstrate that the age and local environmental cage variables were driving the composition of the faecal bacteria and were more deterministically important than the host genotype. These findings have major implications for understanding the significance of functional metagenomic data in experimental studies and beg the question; what is being measured in animal experiments in which different strains are housed separately, nature or nurture?

## Introduction

Emerging evidence of an obesity-associated altered microbiome with the potential to influence caloric extraction from the diet and host energy metabolism [1]–[3] has fuelled a surge in both scientific and public interest in the role of the microbiome in the etiopathogenesis of obesity, with particular interest in the functional properties of the gut microbiota, microbe-host signaling and the possibility of using the microbiome as a therapeutic target. However, evidence also suggests that the relationship between the microbiota and obesity is complex, with contradictory findings relating to the nature of the shift in the relative contributions of phyla to the microbiota composition in obesity, and the question of whether the observed shift in the microbiome is more associated with a high-fat diet than genetically induced obesity *per se* [4]–[9]. Given the complexity of the host-microbiome relationship, it is vital that experimental studies on microbiota composition are well-founded at the most basic level as well as at the high end levels of analytical phenotyping, genotyping and functional ecological analysis.

Several rodent models have been developed to investigate the role of the host's genotype on the development of obesity. One such model is the homozygous Zucker (fa/fa) obese rat, which is characterised by an autosomal recessive mutation of the *fa*-gene, encoding for the leptin receptor. This results in reduced sensitivity to leptin, leading to hyperphagia, obesity and hyperinsulinaemia. In contrast, the heterozygous (fa/+) and homozygous (+/+) Zucker genotypes remain lean as they age and do not develop insulin resistance. Previous analyses of the intestinal microbiota of the Zucker rat found differences between obese and lean strains when the animals were housed according to strain [10]. Therefore, we have designed an experiment to explore the effect of age, genotype, obese/lean phenotype, and cage environment on the evolution and development of the faecal microbiota of the male Zucker rat. We aimed to test the hypothesis that the obese phenotype will result in the evolution of a faecal microbiome and host metabotype distinct from the lean Zucker rats, independent of cage or age. We evaluated this by including each of the three different genotypes in each cage.

## Methods

### Ethics statement

All animal work was carried out in accordance with the U.K. Home Office Animals (Scientific Procedures) Act 1986 under a Project Licence which was approved by the AstraZeneca Ethical Review Committee. The specific protocols described in this paper were also reviewed and approved by the local Departmental Review to ensure that they adhered to the principals of minimising animal suffering. The hypothesis/ethical review study code for the animal study conducted at AstraZeneca was HETP24. The protocol review document was ETP40.

### Animals and sample collection

Three strains of male rat were used in this study, Zucker (fa/fa) obese, heterozygous Zucker lean (fa/+), and Zucker lean (+/+) (n = 6 per strain). The animals were bred on site, (Alderley-Park, AstraZeneca) and housed in a conventional animal room in Techniplast P2000 cages at standard room temperature and humidity on a 12 h∶12 h light:dark cycle. The pups were reared with their mothers until separated at weaning; they were housed as littermates in six cages, each containing one rat from each genotype (n = 3 per cage). The rats in all six cages had different mothers and fathers, and the three rats inside each single cage were littermates. Food (SDS breeding diet RM-3) and water were available *ad libitum* throughout the study. At weekly intervals, from 5 to 14 weeks of age, the animals were transferred to a procedures room, weighed, and placed individually in metabolism cages, for no more than 2 hours, for urine and faeces collection. Samples were collected at the same time of day to remove diurnal effects on profiles. The rats had access to food and water whilst in the metabolism cages. At 14 weeks of age, following urine and faeces collection, animals were rendered insentient by inhalation of a 5∶1 mixture of CO_2_∶O_2_, and a blood sample taken by cardiac puncture into lithium heparin blood syringes. Urine was also collected for metabolite analysis (data not shown, Lees *et al*., in preparation) together with a terminal blood sample. Euthanasia was confirmed by cervical dislocation. Faeces were stored at −40°C prior to 16S rRNA gene profiling analysis.

### Sample preparation

For 16S rRNA gene profiling, four faeces collection time points were selected from the ten time points of the study, when the animals were: five, seven, ten and fourteen weeks of age. The faecal DNA was extracted from at least two different pellets, with a total weight of approximately 200 mg. The Qiagen QIAamp DNA stool kit was used for DNA extraction, as per the manufacturer's instructions, with an additional bead-beating step for homogenisation of sample and lysis of bacterial cells (0.1 g 0.1 mm sterile glass beads, FastPrep bead-beater (Q-BIOgene), setting six (6 metres per second) for 20 seconds, repeated a further two times with 5 minutes on ice between cycles). Following DNA extraction, DNA concentration and purity was determined using a NanoDrop Spectrophotometer (Thermo Scientific, Wilmington, DE, USA), and diluted to a working concentration of 10 ng/µl. The polymerase chain reaction (PCR) was used to amplify the V1-V3 regions of the 16S rRNA gene from each DNA sample using the primers shown in Table S1 and was performed in triplicate on all samples using a C1000 Thermal Cycler (Bio-Rad, USA). PCR mixtures (50 µl) contained *Taq* polymerase (0.25 µl, 5 U/µl solution), buffer (10 µl), MgCl_2_ (3 µl, 1.5 mM), deoxynucleoside triphosphates (dNTPs, 0.4 µl, 0.2 mM of each dNTP), 1 µl of each barcoded primer, 1 µl of each sample DNA (10 ng), and 34.35 µl H_2_O. The PCR cycle conditions were: 95°C for 5 min initial denaturation, 25 cycles of amplification at 95°C denaturation for 30 s, annealing at 55°C for 40 s, and extension of 72°C for 1 min, with a final extension of 72°C for 5 min. PCR products (created in triplicate) were pooled for each sample, and purified using a Qiagen QIAquick PCR purification kit, quantified, again using a NanoDrop Spectrophotometer. The samples were normalised to 5 ng/µl, and 4 µl was transferred to a new micro-centrifuge tube for pooling of samples. The samples were run on three PTPs (Pico Titre Plates), and so were pooled in to three 1.5 ml micro-centrifuge tubes. Samples were sent to the University of Liverpool to be sequenced on a Roche 454 GS FLX sequencer. All sequences are deposited in the European Nucleotide Archive under accession number PRJEB5969.

### Data processing

Samples were processed using the Ribosomal Database Project (RDP) pyropipeline [11] to remove any reads that were less than 250 base pairs, <Q20 and contained any ambiguities (Ns). The filtered sequences were classified using the RDP classifier [12] and the relative proportions of phyla and families calculated. To account for variation in sequence reads per sample, the samples were normalised to the lowest sequence count per animal [13] (Table S2). The resultant relative abundance values were used for multivariate (PCA) and univariate (one-way ANOVA) statistical analysis. UniFrac distances (both unweighted and weighted [14]) were calculated using Mothur v 1.28.1 [13].

### Statistical analysis

UniFrac unweighted distances were analysed by non-metric multidimensional scaling (NMDS) in R [15]. The UniFrac unweighted distances were analysed at each time point using an unpaired Student's *t* test after normality of data had been ensured. Univariate statistical analysis of relative abundance values was performed using GraphPad Prism version 6 software (GraphPad Software, San Diego, CA). To meet the assumptions of the one-way analysis of variance (ANOVA), the data were assessed for normality prior to analysis using the D'Agostino-Pearson test, and the Bartlett's test for equality of variance. The differences between samples from differing time points were assessed using one-way ANOVA and Tukey-Kramer multiple comparisons test. Analysis of the samples at the individual operational taxonomic unit (OTU) level was undertaken in STAMP [16] using genotype, cage and week as the three main discriminators. The means for each OTU were tested using an ANOVA and corrected for multiple testing using the Bonferroni correction. In addition, the data were divided into four time points and tested independently of each other to remove the time factor from the analysis and to allow for the effect of cage and phenotype to be measured at the OTU level.

### Multivariate analysis of relative abundance values

To aid interpretation of the data and quickly visualise trends associated with age, genotype and cage environment, principal component analysis (PCA) was applied to the relative abundance data [17]. The relative abundance values were filtered so that only bacteria detected in at least 75% of animals per group were included in models. PCA was performed on mean-centred, Pareto-scaled [18] data for phylum-level data, using SIMCA 12.0 (Umetrics 2009). For PCA modelling of family-level profiles, data were again mean-centred and a log_10_ transformation was required due to the distribution of the data [19].

## Results

### Metataxonomic characterisation of the faecal microbiota

Data generated from the 16S rRNA gene profiling of faeces from rats aged five, seven, ten and fourteen weeks of age were examined with respect to age- and phenotype-related variation, and also the effects of housing (cage effect) were considered.

### Age-related development of the gut microbiota

**Figure 1:**
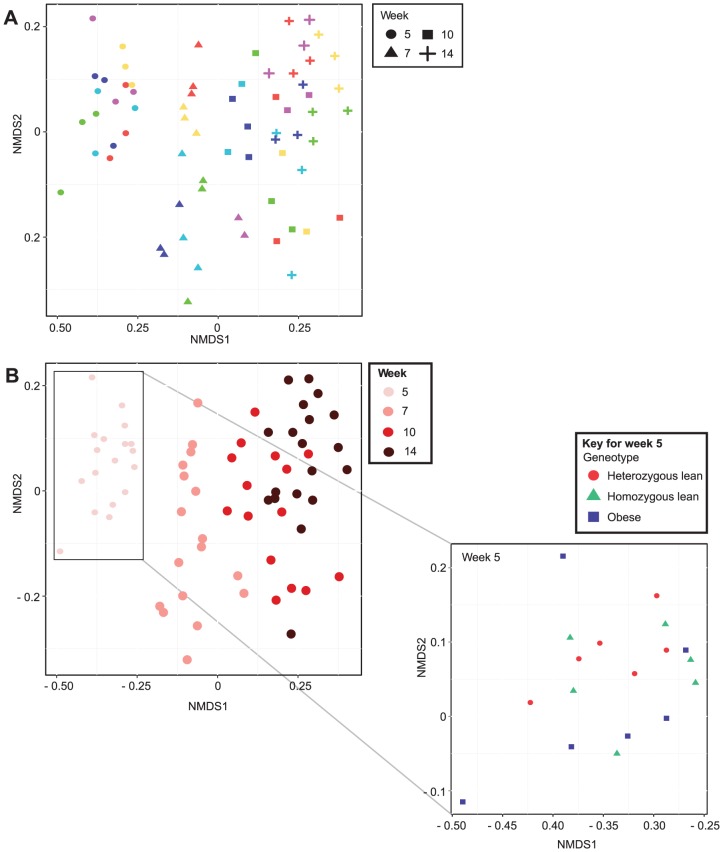
Non-Metric Multidimensional Scaling (NMDS) based on the unweighted UniFrac distances between the faecal samples. A: Samples are coloured by cage (1, red; 2, yellow; 3, green; 4, cyan; 5, dark blue; 6, purple). B: Samples are coloured by the age of the animals at sample collection; the genotype of the animals is shown for week 5. All time points coloured according to genotype are shown in supplementary information (Figure S1).

**Figure 2:**
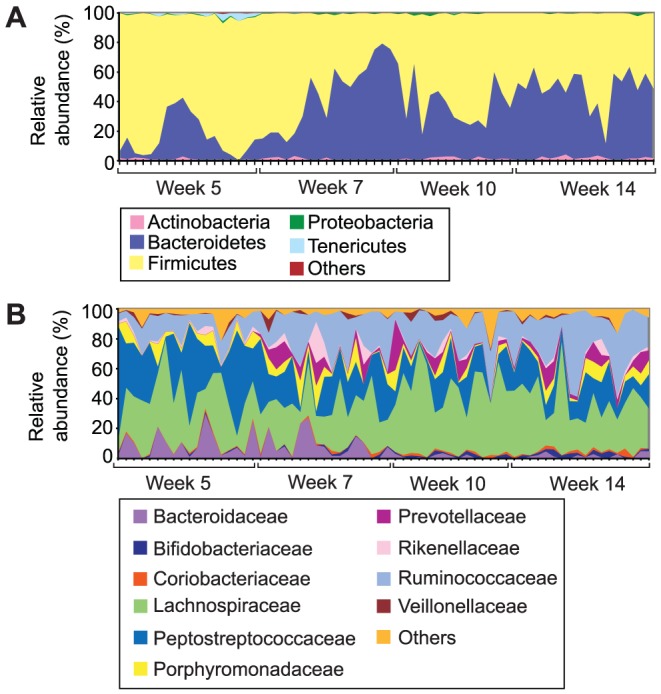
Relative abundances of bacteria across all 68 animal samples ordered by time point. A: Phylum-level; key: ‘Others’ composed of TM7 and *Verrucomicrobia*. B: Family-level; key: ‘Others’ composed of the families: *Alcaligenaceae, Anaeroplasmataceae, Bacillaceae, Clostridiaceae, Enterobacteriaceae, Erysipelotrichaceae, Eubacteriaceae, Halomonadaceae, IncertaeSedis XIII, IncertaeSedis XIV, Lactobacillaceae, Peptococcaceae, Pseudomonadaceae* and *Sphingomonadaceae*. Plot labels: O = obese, L = homozygous lean, H = heterozygous lean; number indicates cage number 1–6.

Based on UniFrac distances (Figure 1) and the 16S rRNA gene profiling of the faecal samples, the intestinal microbiota showed clear age-related trends at the phylum, family and OTU level. At the phylum level there was a decrease in the *Firmicutes*:*Bacteroidetes* ratio (from an average ratio of 5.38 at week five, to 1.05 at week fourteen), with both phyla varying with increasing age (Figure 2A). At the family level, aging in the Zucker rat was associated with a reduction in *Bacteroidaceae* and *Peptostreptococcaceae*, and an increase in *Ruminococcaceae* and *Bifidobacteriaceae* (Figure 2B). Statistical analysis using one-way ANOVA was not appropriate due to the heteroscedasticity of the relative abundance data at both the phylum and family level (when comparing values from differing time points, the variance of the groups differed significantly), as judged by Bartlett's test for equal variances. Transformation of the data failed to resolve this issue. When each dataset was tested across the four time points, 24 OTUs were found to vary significantly due to age (Table S3 and Figure S2). The differences ranged from 15-25% enrichment for OTU001 (*Clostridium* XI (family *Peptostreptococcaceae*)) in week 5 compared to weeks 7, 10 and 14. While OTUs 035 and 051 changed between 0.4 and 0.5% and were enriched in week 14 compared to the other weeks for both OTUs. Seventeen OTUs varied when each time point was analysed independently of each other time point (Table S4 and Figure S3). For week five, 3 OTUs varied between the cages; at week seven, 5 OTUs; at week ten, 3 OTUs; and at week fourteen, 8 OTUs varied. There were no consistent changes in the OTUs between cages. For example, cage 3 at week 5 showed enrichment of OTU017 (genus *Bacteroides* enriched between 10-15% over all other cages) and OTU032 (genus *Subdoligranulum* enriched between 5–6% over all other cages) and for cage 1 at week 5 OTU001 (genus Clostridium XI enriched between 34–52% over all cages) was enriched. Only OTU002 and OTU019 showed any changes from week to week and only OTU019, changed from one to another i.e. week 10 to week 14; however, only some of the cages showed the same change between the two time points. In addition, the age of the animals was the largest source of systematic variation in the PCA models of the phylum and family level data (Figures S4A and S5A).

### Impact of the cage environment

**Figure 3:**
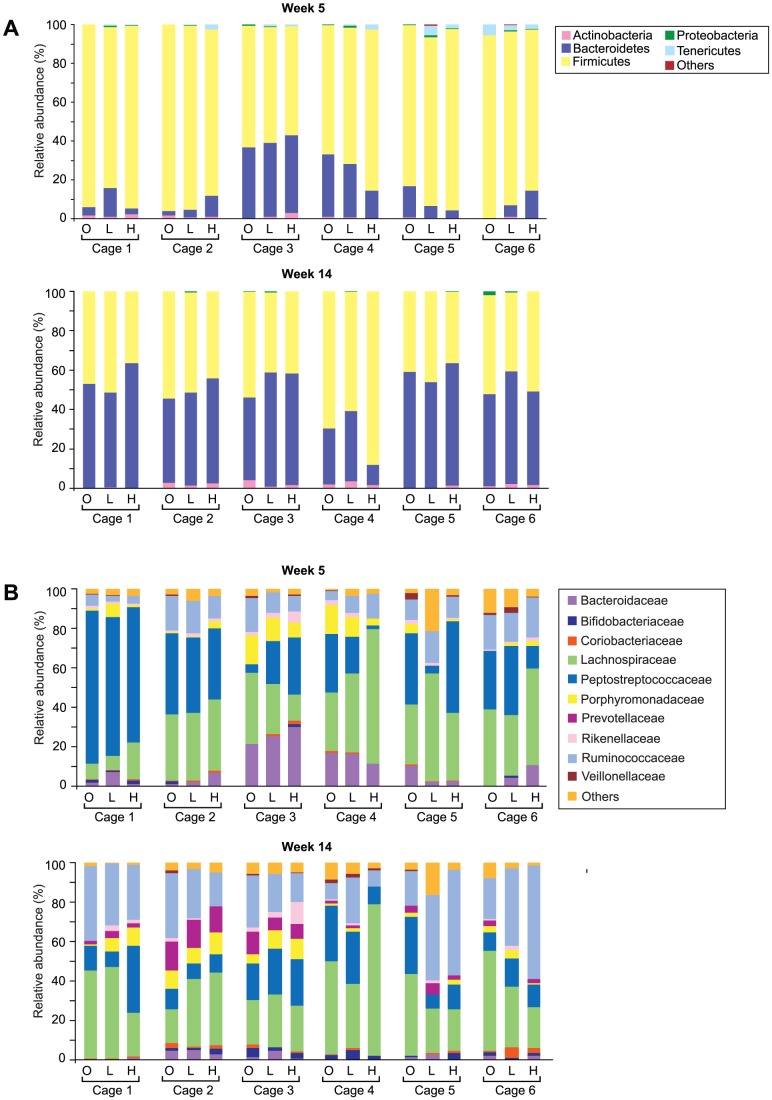
Relative abundances of bacteria for all animals grouped according to cage, at weeks 5 and 14. A: Phylum-level; key: see Figure 2 legend. B: Family-level; key: see Figure 2 legend. Data for weeks 7 and 10 are shown in Figure S9 (phylum) and S10 (family). Key: O = obese, L = homozygous lean, H = heterozygous lean.

**Figure 4:**
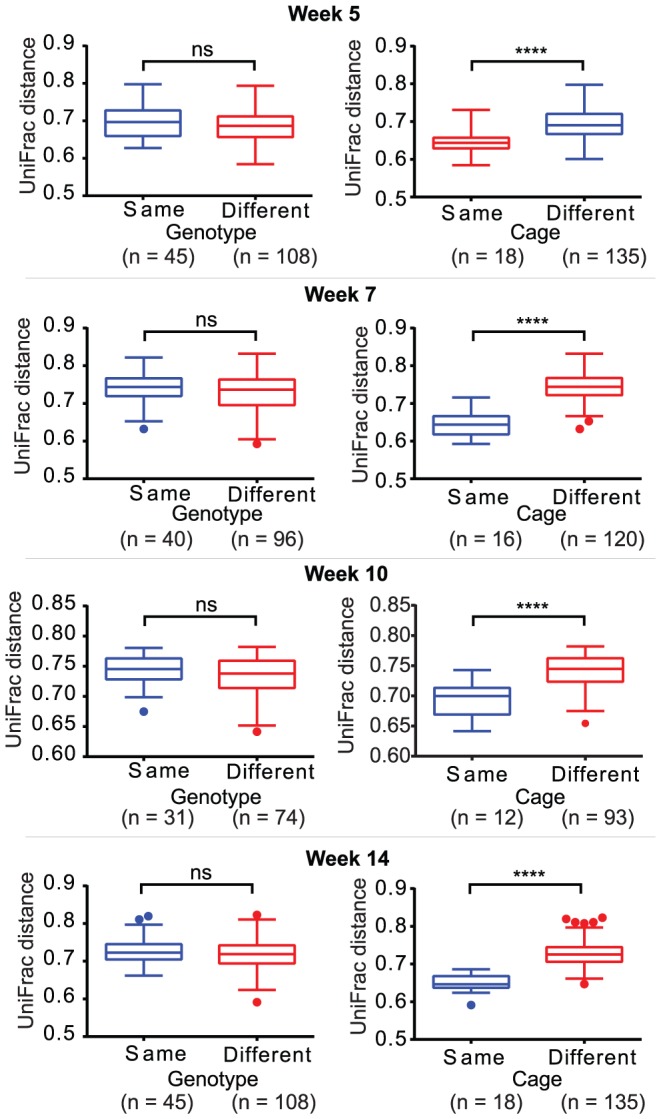
Box plots of the unweighted UniFrac distances. Box plots showing the median, lower and upper quartiles of the unweighted UniFrac distances at each time point comparing the effect of genotype and cage on the community structure. Whiskers were calculated using the Tukey method; filled circles represent outliers. A lower UniFrac distance indicates greater similarity between two microbial communities (Student's *t* test: ns = not significant; asterisks indicate significant differences: ****P<0.0001).

The intestinal bacteria profiles of animals from within the same cage exhibited similarities at the phylum and family level, in spite of the differing obese and lean phenotypes present within each cage. In the taxon-based analysis, cage environment-associated trends in the phylum and family-level datasets were not obvious when all time points were considered together (Figures S4C and S5C), as age at sample collection was the dominant source of systematic variation, and obscured any cage-associated trends. However, there was evidence of cage-environment associated trends, at both the phylum and family-level, when each timepoint was considered independently (Figure 3, Figure S6 and S7). Cage-associated clustering of samples was also evident in the NMDS plot based on the unweighted UniFrac distances between faecal samples (Figure 1). The mean unweighted UniFrac distances of animals from within the same cage were significantly lower (P<0.0001) than animals from differing cages at each time point (Figure 4), and significant differences between cohoused and non-cohoused animals were also observed in the weighted UniFrac distances at week 5 (P<0.001), week 7 (P<0.0001) and week 14 (P<0.01) (Figure S8). The effect of animal housing was most prominent at the beginning of the study in samples from animals at five and seven weeks of age, but differences persisted until the end of the study (Figures S9 and S10). Significant differences were found in the relative abundances of *Bacteroidetes* and *Firmicutes* at the phylum level, and *Bacteroidaceae*, *Lachnospiraceae*, *Peptostreptococcaceae*, *Porphyromonadaceae, Prevotellaceae* and *Ruminococcaceae*, at the family level, between the cages at weeks 5, 7 and 14 (P<0.05) (Table S5 and Table S6), with cages three and four showing significantly higher *Bacteroidetes* at week 5; cages one and two showing significantly higher *Firmicutes* at week 7; and cage four showing significantly higher *Firmicutes* at week 14, compared to all other cages. At the OTU level, only OTU061 was different between cages (corrected P-value = 0.036) across all time points. This OTU was found to be enriched in cage 3 when compared to cages 2, 4, 5 and 6 and clusters in the genus *Bifidobacterium* (Figure S11).

### Phenotypic variation in the faecal microbiota

**Figure 5:**
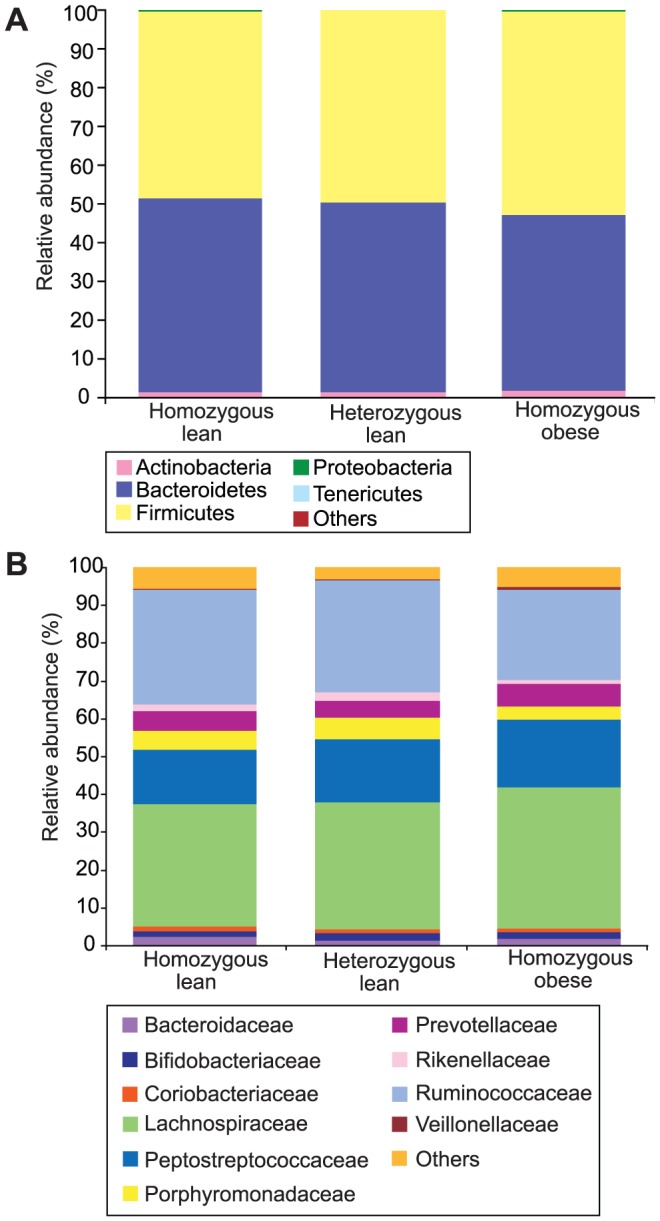
Mean relative abundances of bacteria for each genotype at week 14 (n = 6 per genotype). A: Phylum level; key: see Figure 2 legend. B: Family level; key: see Figure 2 legend. Mean relative abundances of each phylum and family for each genotype at each time point (weeks 5, 7, 10 and 14) are shown in Figure S15 (phylum) and S16 (family).

Food was available *ad libitum* and, despite exhibiting the normal weight-gain-associated-phenotypes expected for these animals (Figure S12 and S13), both multivariate and univariate statistical analyses of the relative abundance values at the phylum, family and OTU levels for samples across all time points, and each timepoint separately, found no differences between the lean and obese phenotypes (Figure 5, Figures S4B and S5B). No statistically significant differences (P<0.05) were found in the relative abundance values of bacterial phyla and families between the three genotypes, except in the relative abundance of *Proteobacteria*, which was higher in samples from homozygous lean animals at week 5 (Figure S14). In the phylogenetic analysis, the NMDS plot based on the unweighted UniFrac distances failed to show any clear genotype-based clustering of samples at any of the time points (Figure S1). No differences were found when comparing the mean unweighted (Figure 4) or weighted (Figure S8) UniFrac distances from animals of the same and different genotypes.

## Discussion

In this study, the age of the rats was found to be the most significant source of systematic variation in the faecal bacterial profile analyses at the phylum, family and OTU levels. Cohabitation had a significant impact on the intestinal microbiota, with more similar communities derived from co-housed animals. The impact of differences in host genotype and phenotype were largely undetected.

The predominant phyla detected in the faecal samples of the Zucker rats in this study were *Firmicutes* and *Bacteroidetes*, with significantly lower detection of *Actinobacteria* and *Tenericutes*; this is consistent with previous analyses of faecal bacterial profiles from rats [20], [21], mice [22]–[24], and humans [1], [3], [25], [26]; although certain studies have seen much greater representation of bacteria from the *Actinobacteria* phylum in humans [27], [28], mice [8] and rats [29] and the *Proteobacteria* phylum in rats [29]. Interestingly, the average relative abundance of *Tenericutes* exceeded that of *Proteobacteria* in samples from animals at five weeks old, in contrast to other analyses of rat faecal microbiota [30], [31]. The observed actinobacterial variability may be due to the primers used for the PCR [32] or the DNA extraction kit used [33], and it is important to note that the hypervariable region of the 16S rRNA gene we selected to amplify (V1-V3) may underestimate the contribution of *Bifidobacteria* to the faecal bacterial profile [34].

At the phylum level, the most significant age-related trend was a decrease in the *Firmicutes*:*Bacteroidetes* ratio with increasing age, in contrast to the findings of previous investigators [8], [35]. Given that the ages of the rats, 5–14 weeks, is more representative of maturation than aging *per se*, it is likely that the age-related trends observed here in the Zucker rat reflect normal development of the microbiota towards a stable climax community. The composition of the intestinal microbiota is known to vary throughout infancy to adulthood, with further variation described in the elderly [36]–[38]. The increasing use of culture-independent direct sequencing techniques will facilitate our understanding of precisely how the intestinal microbiota varies with age, but these results demonstrate the significance of age on the composition of the intestinal microbiota and the importance of the consideration of this variable in the context of designing and interpreting animal studies.

No significant differences were found between the intestinal bacteria profiles of the three Zucker rat genotypes at either the phylum or the family level in the taxon-based analyses, and bacterial communities from the same genotype were not found to be more similar than communities from animals of differing genotypes when the UniFrac distance measures were compared. This result is interesting in light of the attention given to the possibility of an obesity-associated altered microbiome, with an increased potential for energy harvest [1]–[3], and also considering the clear phenotype-based differentiation observed in the ^1^H NMR spectroscopy-based metabolite profiles of the urine, plasma and tissues of these animals (Lees *et al*., in preparation).

In a previous study of the faecal bacterial profiles of the Zucker rat, employing DGGE and fluorescence *in situ* hybridization, differences between all three strains of the Zucker rat were observed, in spite of no phenotypic difference between the two lean strains. It was proposed that the microbiotal differences between the two lean strains were due to host genotype influence on the composition of the faecal microbiota [10]. However, in contrast to the present study, the animals were housed according to genotype, thus the cage environment (and coprophagic activity of the animals) is likely to have been influential in the experimental outcomes and may have reinforced or potentially enhanced any differences.

Certain studies have alluded to a more complex involvement of the microbiota in obesity than perhaps first indicated [4] and the nature of the shift in the relative contributions of phyla to the microbiota composition in obesity has also been contested [5]. Additionally, there is gathering support for the role of diet, rather than obesity itself, in altering bacterial profiles, with shifts in the intestinal microbiome found to be associated with a high-fat diet rather than genetically induced obesity [4], [6]–[8], [39], [40]. With these studies in mind, it is perhaps unsurprising that a quantitative difference in chow consumption, as would be expected between the obese and lean phenotypes analysed here [41]–[45], did not result in a difference in bacterial profiles between the obese and two lean phenotypes. Nevertheless, a more recent analysis of the leptin-resistant *db/db* mouse model identified compositional differences in the gut microbiota between the genetically obese and lean mice [46]; although, again it is unclear to what extent the arrangement of animal housing contributed to these results.

Several studies have explored the regulation of the intestinal microbiota by both host genes and the microenvironment in rodents [7], [47]–[50]. In a quantitative PCR-based analysis of several germfree inbred strains of mice colonised with altered Schaedler flora (ASF), the microenvironment was found to influence the intestinal microbiota, with animals in differing cages showing divergence in ASF profiles. However, cohabitation of differing inbred strains of mice preserved most of the interstrain variation, with species variation in coprophagic behaviour suggested as a potential cause [49]. Further to this, Dimitriu and colleagues found that the response of faecal bacteria profiles to cohousing was strongly dependent on mouse genotype, with immunodeficient mice being more resistant to bacterial colonisation than wild type mice [51]. Similarly, Campbell and colleagues found host genetics to significantly correlate with bacterial phylotypes. Cohabitation of different strains revealed an interaction between host genetic and environmental factors, with bacterial communities more similar between co-housed animals, but with strain specificity maintained [50]. However, in a study of five common laboratory mouse strains, caging was found to contribute more variance to the murine microbiota composition than variation in genetics (31.7% compared to 19%, respectively), but inter-individual variance was the largest contribution (45.5%) [7]. Here, the intestinal bacteria profiles of animals from within the same cage showed clear similarities at the phylum and family level in the taxon-based analysis, in spite of the differing genotypes/phenotypes present. Additionally, comparison of UniFrac distances demonstrated that rats co-housed had significantly more similar bacterial communities than animals from different cages.

The obese and lean Zucker rats from within the same cage shared the same mother and the same cage environment from an early age and throughout the study. The maternal microbiota has been shown to be a significant indicator of offspring microbiota composition, irrespective of genetic background, resulting in similarities between progeny despite strain differences [52]. Furthermore, a study comparing knock-out mice, deficient in Toll-like receptors, with wild type animals, found that this genetic difference had a minimal impact on the composition of the microbiota, and that familial transmission of the maternal microbiota was the dominant source of variation in progeny microbiota composition [53]. The inheritance of the microbiota was also shown by Ley and colleagues in lean and *ob/ob* mice at the genus level; however, phylum-level distinctions between the two phenotypes were also observed [22], indicating that phenotypic differences may dominate in certain circumstances.

In addition to the influence of the maternal microbiota on the intestinal bacteria of offspring, the immediate cage environment has been shown to be a highly influential factor in microbiota development [52], [54] and cohousing of litters will likely have reinforced inter-cage differences in the bacterial profiles of the Zucker rats. Rodents are coprophagic and ingestion of phenotypically differing littermates' faeces will have occurred from an early age, contributing towards the development of a common microbiome in animals occupying the same cage [55]. The influence of the cage environment on the developing intestinal microbiome was clearly demonstrated by Friswell and colleagues; marked changes were observed in the gut microbiota of mice re-located to new housing at four weeks of age, but not when mice were re-located at eight weeks of age [52]. Additionally, Ma and co-workers found that relocation of mice to new cages in a different intracampus facility was associated with transient variation in the composition of the faecal microbiota [53]. Furthermore, the effect of cage-environment has proved significant in a previous analysis of bacterial recolonisation profiles in rats following antibiotic exposure [56].

Germ free animal models have also been utilised to understand the contributions of various factors to the development of the microbiome; in a comparison of germ free mice either gavaged with a microbiota harvested from adult wild type mice, or allowed to acquire an intestinal microbiome from the cage microenvironment, authors found that the cage microenvironment mitigated the effects of the founding community [54]. More recently, a study of germ-free mice gavaged with the cultured microbiota of a twin pair discordant for obesity, demonstrated the significant impact of within-cage coprophagy on host metabolism. Recipients of the obese and lean microbiotas were co-housed, leading to certain bacterial species successfully invading the microbiome of co-housed animals, an effect that was diet dependent [57].

A potential limitation of our study is the lack of accurate measurement of food intake, prohibited by the complex nature of the animal housing design, which might have further strengthened our conclusions. However, we are satisfied our assumptions are reasonable, due to previous studies in our facility and a number of publications detailing the relative food intake of obese and lean Zucker rats of the same approximate age and bodyweight. Thus, obese Zucker rats, fed *ad libitum*, were found to have an increased food intake of between 30–60%, compared to the lean animals [58]–[60]. Additionally, we acknowledge that the use of 454 technology, and level of sequencing employed here, will have broadly characterized the samples in terms of the major patterns of variation, and that less abundant species of the populations sampled may not have been represented.

## Conclusions

This study presents novel findings relating to how the faecal microbiota in the Zucker rat develops with age through juvenile, pubertal and post-pubertal stages. In addition, these results clearly demonstrate the significance of both age and cage environment on the composition of the faecal microbiota, in the context of an obese animal model, with both variables exerting a greater pressure on intestinal microbiota community structure than obese or lean phenotype and chow consumption.

In the context of the recent explosion of research into the compositional and functional aspects of the intestinal microbiota, these data emphasise the need to control for the effect of the microenvironment on the intestinal microbiome. As a minimum requirement, researchers need to be transparent regarding the specific animal housing arrangements when publishing studies, to allow for informed interpretation of data. This may be particularly important in studies whereby group-housing of animals according to genotype/phenotype acts to positively reinforce a particular compositional or functional aspect of the intestinal microbiota, effectively amplifying any differences between groups in differing cages. The profound effects of the housing of experimental animals on outcomes demonstrated here have clear implications for investigations relating to the development of the intestinal microbiota, and to microbiome-host co-metabolism, and should be given greater attention when designing studies.

## Supporting Information

Figure S1 **Non-Metric Multidimensional Scaling (NMDS) based on the unweighted UniFrac distances between the faecal samples.** Central plot shows samples coloured according to animal cage (1–6), with sample marker shape representing time of sample collection. Enlarged plots show the genotype of the animals at each sample collection time point.
(DOCX)

Figure S2 **ANOVA of the means of OTUs, demonstrating that several OTUs varied between different time points across all the animals tested.**
(DOCX)

Figure S3 **ANOVA of the means of OTUs, demonstrating that several OTUs varied between cages at each time point.**
(DOCX)

Figure S4 **PCA scores plots generated using relative abundance values of the three most abundant phyla:** ***Bacteroidetes*** **,** ***Firmicutes*** **and** ***Actinobacteria*** **, in samples collected from all animals at all time points (mean centred, Pareto-scaled data; R^2^ = 0.99, Q^2^ = 0.96).** Principal components 1 and 2 (PC1 and PC2) are shown with the percentage of explained variance described by each component. A: Samples are coloured according to the age (in weeks) at which the sample was collected. B: Samples are coloured according to the genotype of the animal. C: Samples are coloured according to the cage (1–6) of each animal. The scores plot in (A) can be used as a reference for the sample time points; the time points are not shown in (B) and (C) to aid visualisation of potential trends.
(DOCX)

Figure S5 **PCA scores plots generated using relative abundance values of the six most abundant families:** ***Bacteroidaceae, Porphyromonadaceae, Rikenellaceae, Lachnospiraceae, Ruminococcaceae*** **and** ***Peptostreptococcaceae*** **, in samples collected from all animals at all time points (Log_10_ transformed, mean centred data; R^2^ = 0.83, Q^2^ = 0.01).** Principal components 1 and 3 (PC1 and PC3) are shown with the percentage of explained variance described by each component. A: Samples are coloured according to the age (in weeks) at which the sample was collected. B: Samples are coloured according to the genotype of the animal. C: Samples are coloured according to the cage (1–6) of each animal. The scores plot in (A) can be used as a reference for the sample time points; the time points are not shown in (B) and (C) to aid visualisation of potential trends.
(DOCX)

Figure S6 **PCA scores plots generated using relative abundance values of the three most abundant phyla:** ***Bacteroidetes*** **,** ***Firmicutes*** **and** ***Actinobacteria*** **.** Plots are shown for samples collected from all animals at weeks 5, 7, 10 and 14 (mean centred, Pareto-scaled data; Week 5: R^2^ = 1.00 Q^2^ = 0.92; Week 7: R^2^ = 1.00 Q^2^ = 0.98; Week 10: R^2^ = 1.00 Q^2^ = 0.97; Week 14: R^2^ = 1.00 Q^2^ = 0.95). In each plot principal components 1 and 2 (PC1 and PC2) are shown with the percentage of explained variance described by each component. Samples are coloured according to the cage (1–6) of each animal.
(DOCX)

Figure S7 **PCA scores plots generated using relative abundance values of the six most abundant families:** ***Bacteroidaceae, Porphyromonadaceae, Rikenellaceae, Lachnospiraceae, Ruminococcaceae*** **and** ***Peptostreptococcaceae*** **.** Plots are shown for samples collected from all animals at weeks 5, 7, and 10 (Log_10_ transformed, mean centred data; Week 5: R^2^ = 0.87 Q^2^ = 0.53; Week 7: R^2^ = 0.82 Q^2^ = 0.06; Week 10: R^2^ = 0.78 Q^2^ = 0.29). In each plot principal components 1 and 2 (PC1 and PC2) are shown with the percentage of explained variance described by each component. Samples are coloured according to the cage (1–6) of each animal. Week 14 is not shown here, as the Q^2^ was negative with the first component, and was thus not considered a valid model.
(DOCX)

Figure S8 **Box plots showing the median, lower and upper quartiles of the weighted UniFrac distances at each time point comparing the effect of genotype and cage on the community structure.** Whiskers were calculated using the Tukey method; filled circles represent outliers. A lower UniFrac distance indicates greater similarity between two microbial communities (Student's *t* test: ns = not significant; asterisks indicate significant differences: ** P<0.01; *** P<0.001; ****P<0.0001).
(DOCX)

Figure S9 **Relative abundances of bacteria at the phylum-level for all animals grouped according to cage, at each time point separately.** Key: O = obese, L = homozygous lean, H = heterozygous lean. Phylum key: ‘Others’ composed of TM7 and *Verrucomicrobia*.
(DOCX)

Figure S10 **Relative abundances of bacteria at the family-level for all animals grouped according to cage, at each time point separately.** Key: O = obese, L = homozygous lean, H = heterozygous lean. Family key: ‘Others’ composed of the families: *Alcaligenaceae, Anaeroplasmataceae, Bacillaceae, Clostridiaceae, Enterobacteriaceae, Erysipelotrichaceae, Eubacteriaceae, Halomonadaceae, Incertae Sedis XIII, Incertae Sedis XIV, Lactobacillaceae, Peptococcaceae, Pseudomonadaceae* and *Sphingomonadaceae*.
(DOCX)

Figure S11 **ANOVA of the means of the OTU061 shows that this OTU was the only one to vary at any significant levels between cages across the 4 time points.**
(DOCX)

Figure S12 **Body weights for each animal at 4 weeks (pre-study) and at every urine sample collection point (weeks 5 to 14).** (A) obese (fa/fa) animals, (B) lean (+/+) animals and (C) lean (fa/+) animals. Colour of data points indicates cage number of the animal.
(DOCX)

Figure S13 **Body weights for each strain at each week including pre-study (at four weeks for age), data expressed as mean ± standard error of the mean.** Asterisks indicate significant differences (one-way ANOVA, followed by Tukey-Kramer multiple comparisons test, * P<0.05; ** P<0.01; *** P<0.001; **** P<0.0001). Green asterisks relate to the comparison of (fa/fa) and (+/+); red asterisks relate to the comparison of (fa/fa) and (fa/+).
(DOCX)

Figure S14 **Box plots of the relative abundance of** ***Proteobacteria*** **for each genotype at each time point.** The median, lower and upper quartiles are shown. Whiskers were calculated using the Tukey method; filled circles represent outliers. Asterisks indicate significant differences (one-way ANOVA, followed by Tukey-Kramer multiple comparisons test, * P<0.05; ** P<0.01; *** P<0.001).
(DOCX)

Figure S15 **A: mean relative abundances of each phylum for each genotype (all time points included). B: mean relative abundances of each phylum for each genotype at each time point separately.** Phylum key: ‘Others’ composed of TM7 and *Verrucomicrobia*.
(DOCX)

Figure S16 **A: mean relative abundances of each family for each genotype (all time points included). B: mean relative abundances of each family for each genotype at each time point separately.** Family key: ‘Others’ composed of the families: *Alcaligenaceae, Anaeroplasmataceae, Bacillaceae, Clostridiaceae, Enterobacteriaceae, Erysipelotrichaceae, Eubacteriaceae, Halomonadaceae, Incertae Sedis XIII, Incertae Sedis XIV, Lactobacillaceae, Peptococcaceae, Pseudomonadaceae* and *Sphingomonadaceae*.
(DOCX)

Table S1 **Primers used to amplify the V1-V3 regions of the 16S rRNA gene.** The unique barcode for each sample is shown in red, and allowed for multiplexing of the samples on the 454 sequencer on three different PTPs (Pico Titre Plate 8ths, 1 2 or 3).
(DOCX)

Table S2 **Sequence counts per sample.**
(DOCX)

Table S3 **The OTUs identified by STAMP to be significantly altered in the faecal samples when grouped by week.** All the means for each group were compared using an ANOVA and multiple testing using the Bonferroni correction (see Figure S2 for more detail).
(DOCX)

Table S4 **OTUs which were significantly changed at each time point between cages (P<0.05, corrected for multiple testing), see Figure S3 for more detail.**
(DOCX)

Table S5 **Significant differences in the relative abundances of** ***Bacteroidetes*** **and** ***Firmicutes*** **between cages (no other phyla were found to be significantly different).** Level of significance: * P<0.05; ** P<0.01; *** P<0.001. Difference between means of cages assessed using one-way ANOVA, followed by Tukey-Kramer multiple comparisons test.
(DOCX)

Table S6 **Significant differences in the relative abundances of families between cages (no other families were found to be significantly different).** Level of significance: * P<0.05; ** P<0.01; *** P<0.001. Difference between means of cages assessed using one-way ANOVA, followed by Tukey-Kramer multiple comparisons test. Univariate statistical comparison of all cages was not possible at week 10 due to the small sample numbers per cage (n<3).
(DOCX)
