# Supplementary material for: Age and Microenvironment Outweigh Genetic Influence on the Zucker Rat Microbiome
Source: PLoS One. 2014 Sep 18;9(9):e100916. doi: 10.1371/journal.pone.0100916 (PMC4169429; doi:10.1371/journal.pone.0100916)
Supplement: Figure S1 — Non-Metric Multidimensional Scaling (NMDS) based on the unweighted UniFrac distances between the faecal samples. Central plot shows samples coloured according to animal cage (1–6), with sample marker shape representing time of sample collection. Enlarged plots show the genotype of the animals at each sample collection time point. (DOCX) [file pone.0100916.s001.docx]

**Figure S1**: Non-Metric Multidimensional Scaling (NMDS) based on the unweighted UniFrac distances between the faecal samples. Central plot shows samples coloured according to animal cage (1-6), with sample marker shape representing time of sample collection. Enlarged plots show the genotype of the animals at each sample collection time point.
