# Supplementary material for: Age and Microenvironment Outweigh Genetic Influence on the Zucker Rat Microbiome
Source: PLoS One. 2014 Sep 18;9(9):e100916. doi: 10.1371/journal.pone.0100916 (PMC4169429; doi:10.1371/journal.pone.0100916)
Supplement: Figure S3 — ANOVA of the means of OTUs, demonstrating that several OTUs varied between cages at each time point. (DOCX) [file pone.0100916.s003.docx]

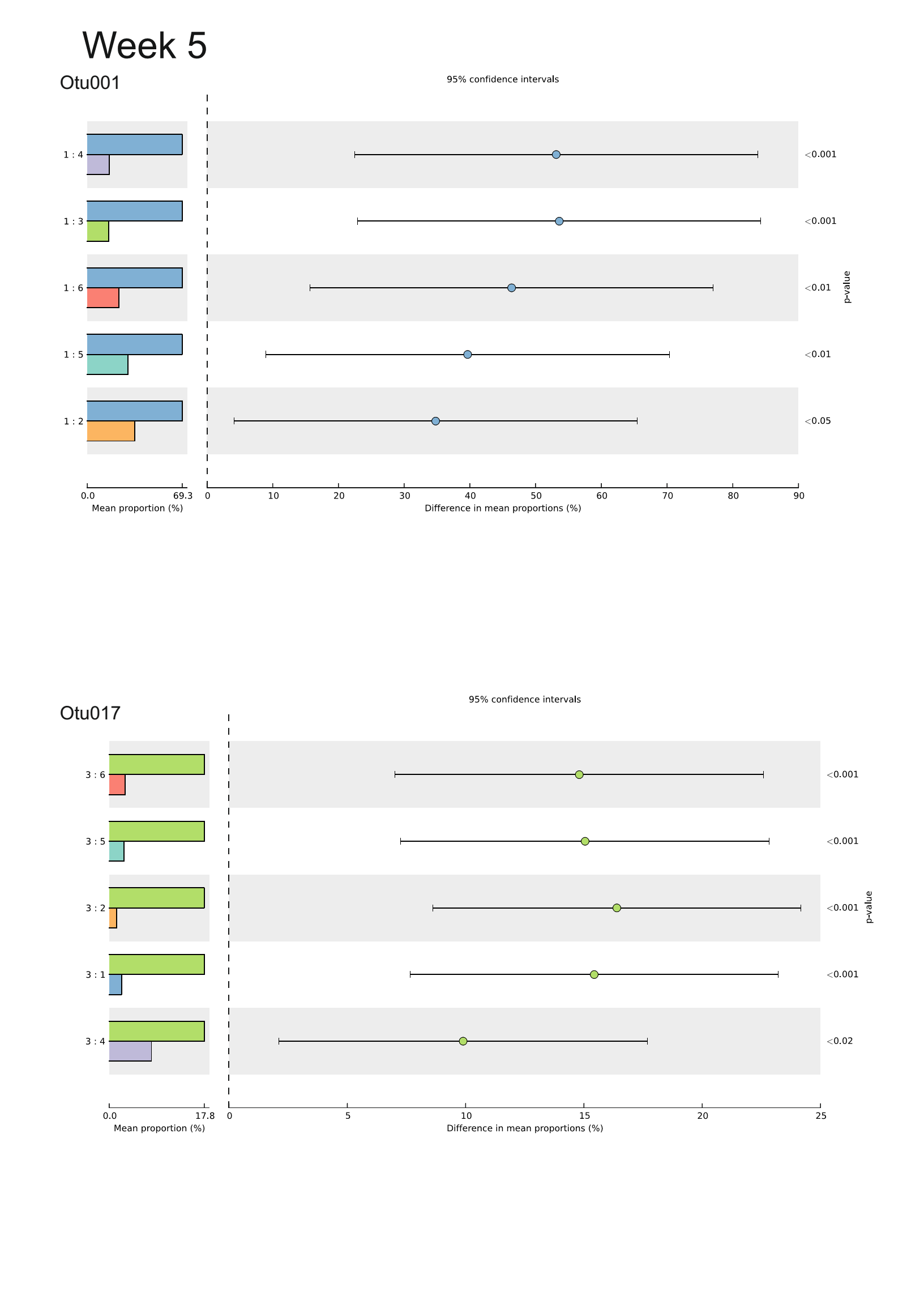


**Figure S3**


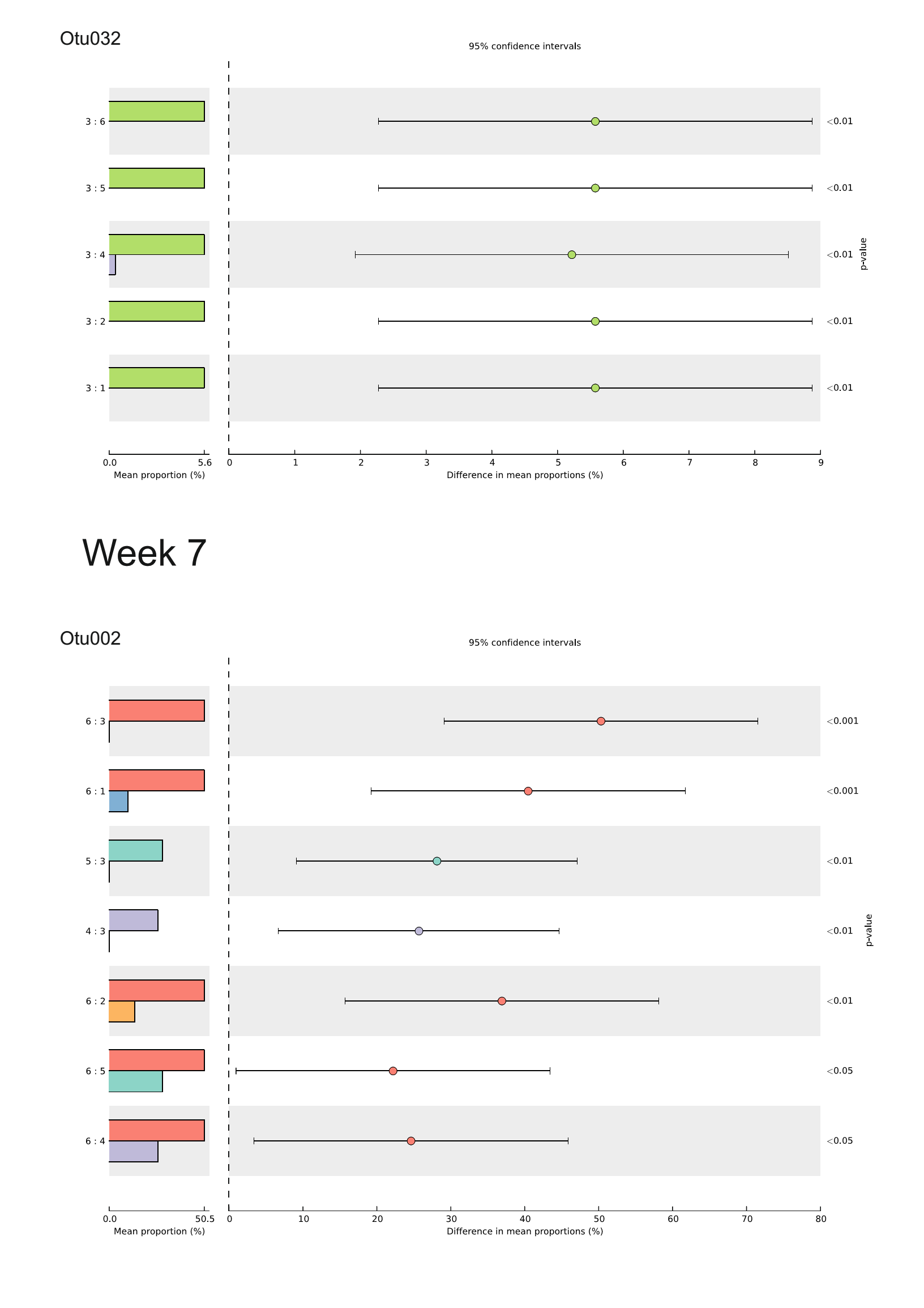


**Figure S3 continued**


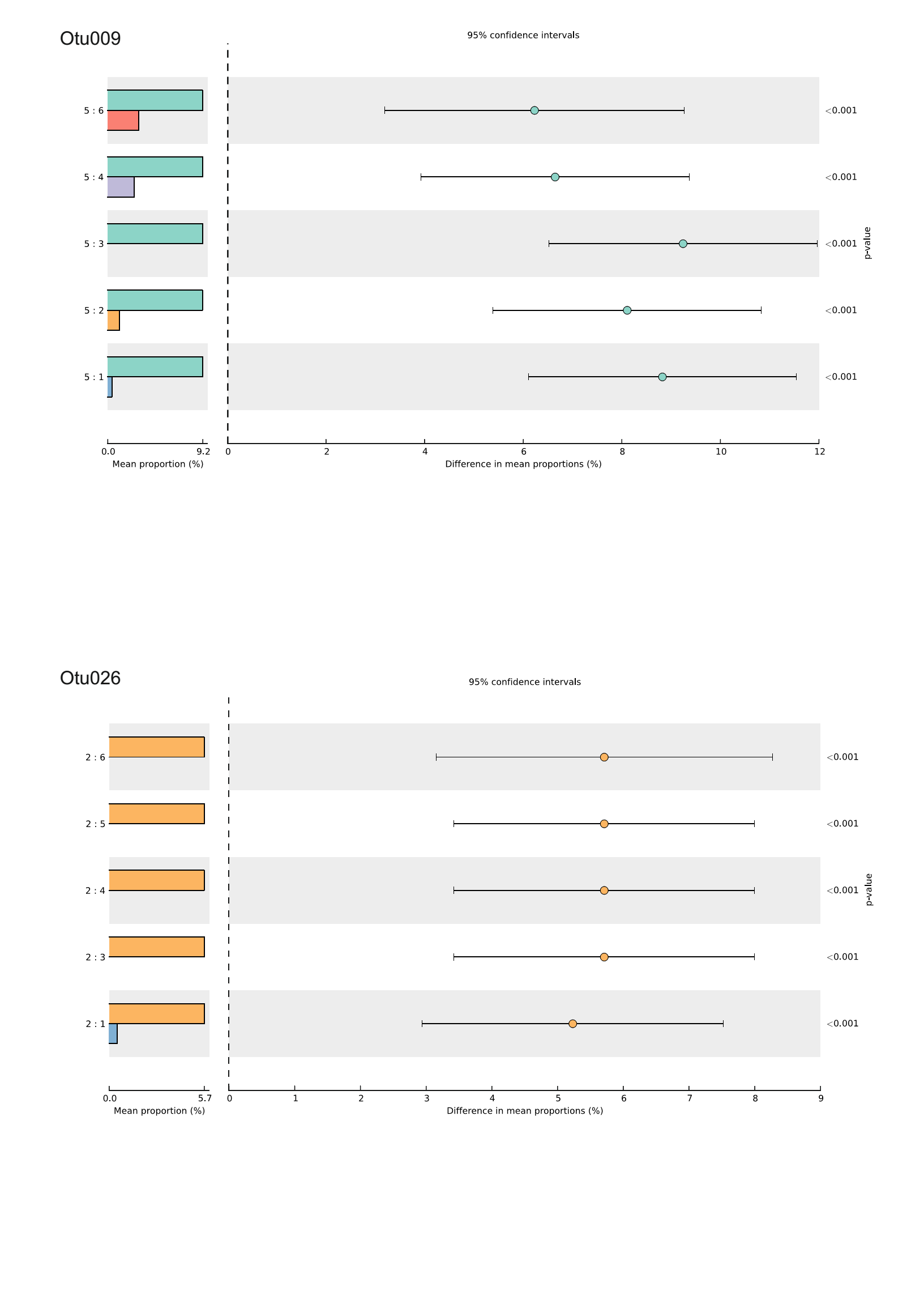


**Figure S3 continued**


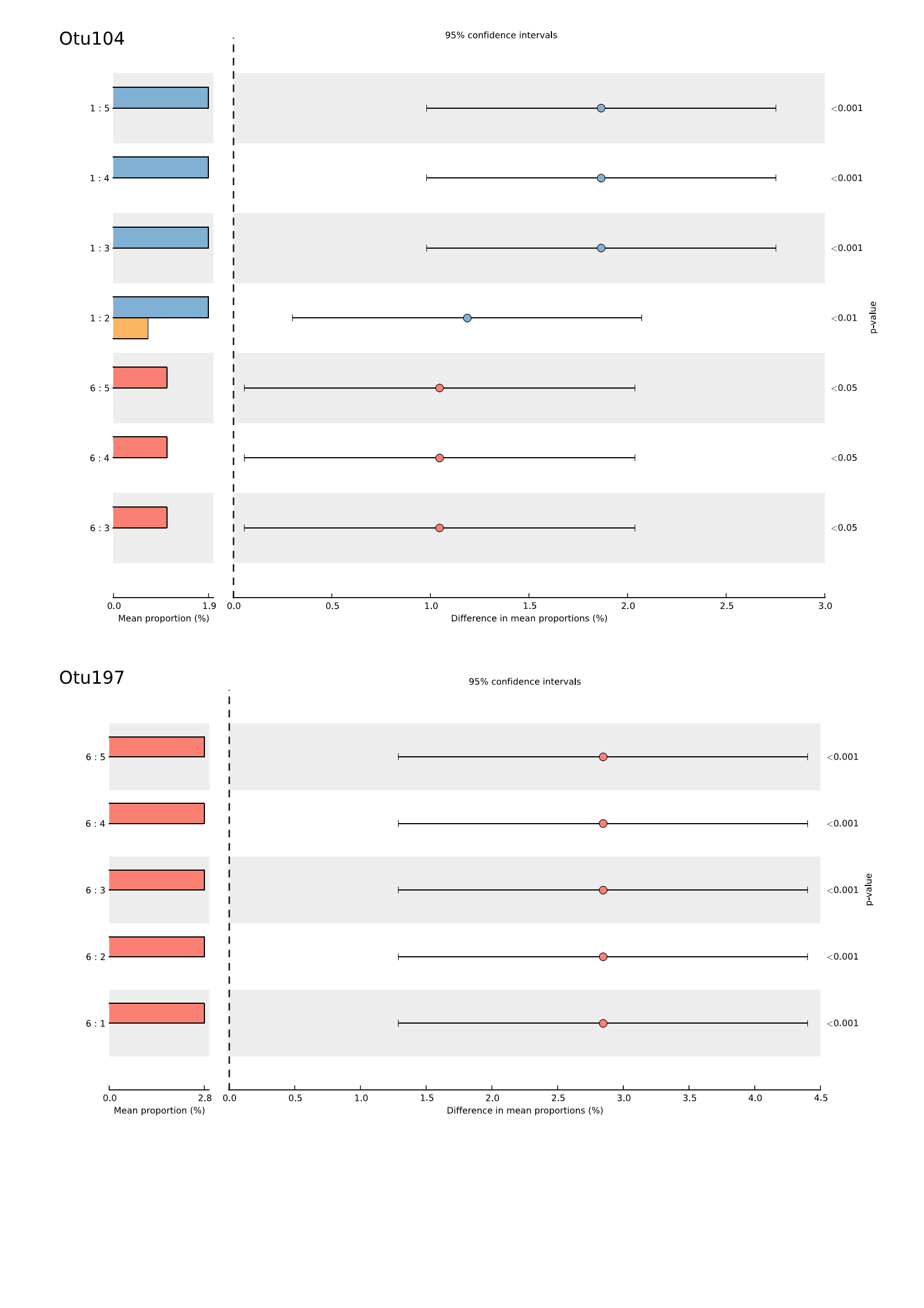


**Figure S3 continued**


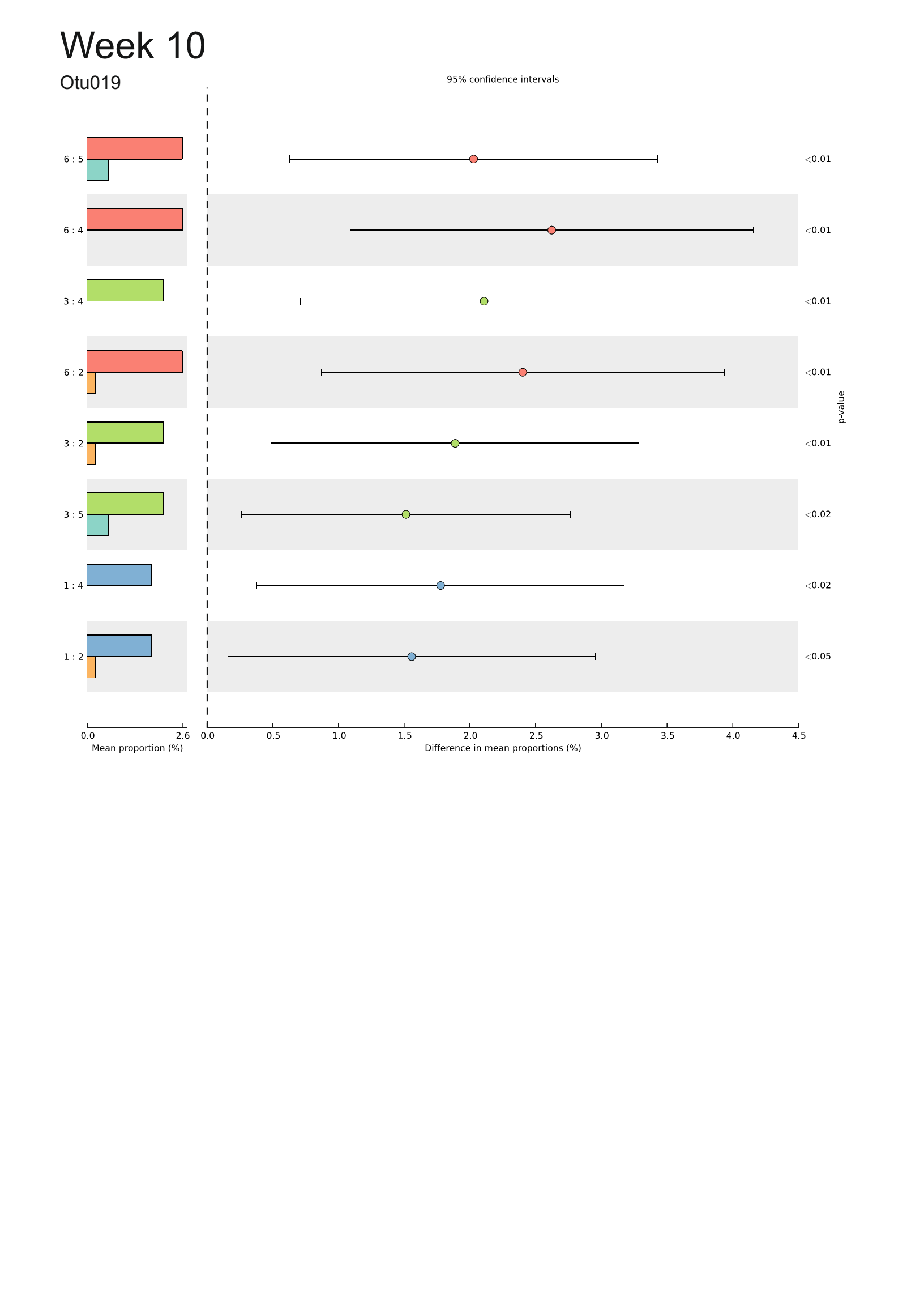


**Figure S3 continued**


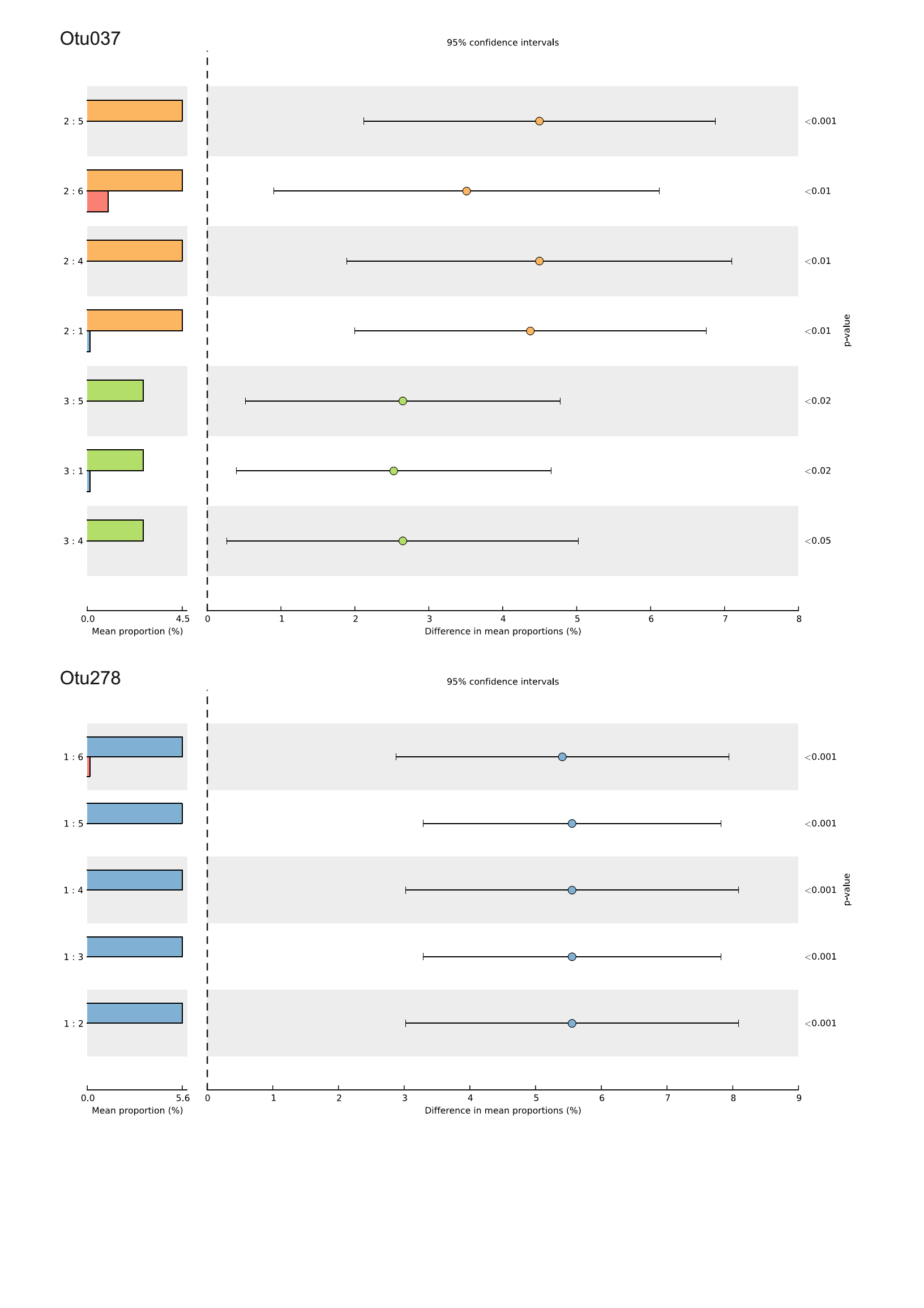


**Figure S3 continued**


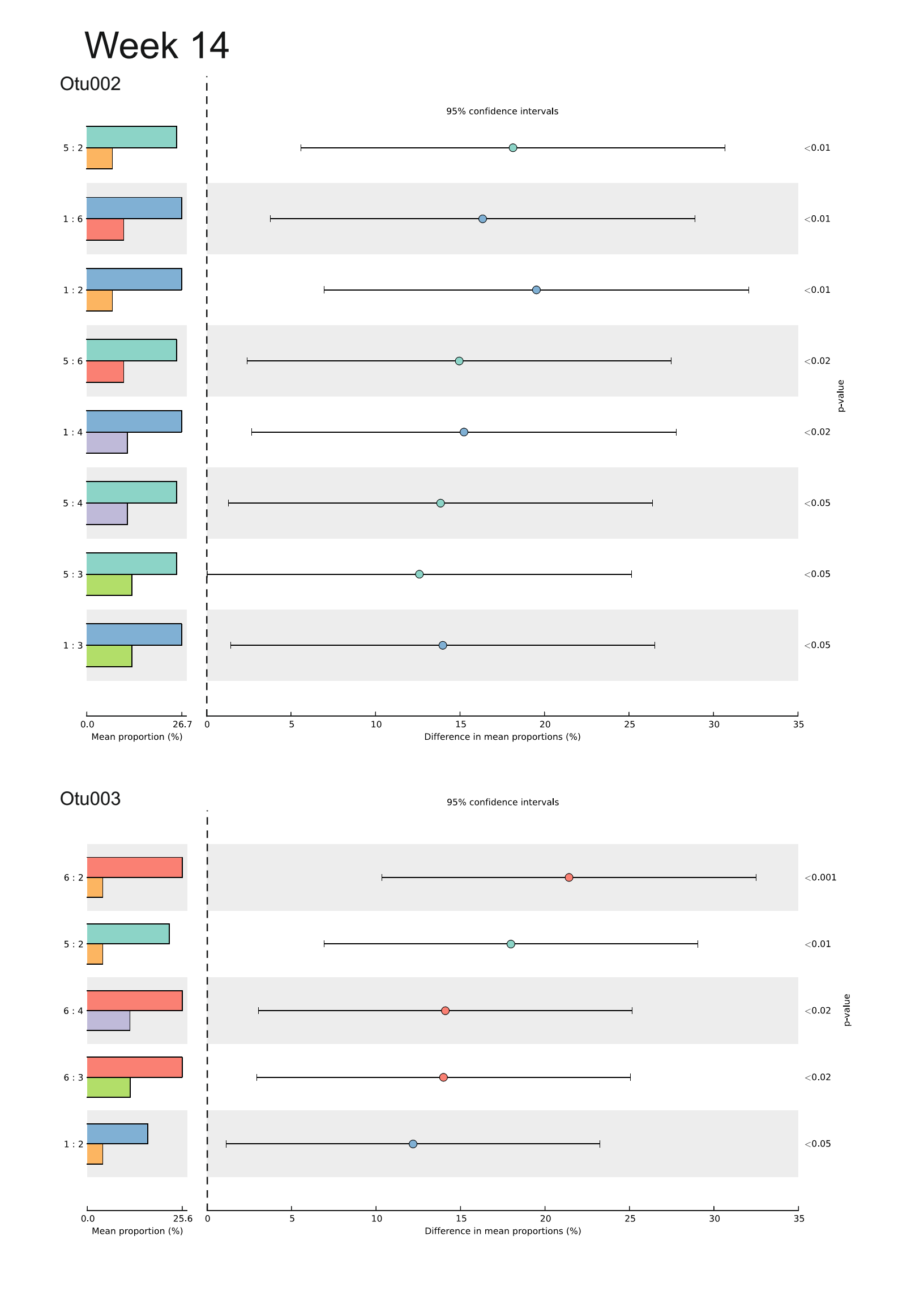


**Figure S3 continued**


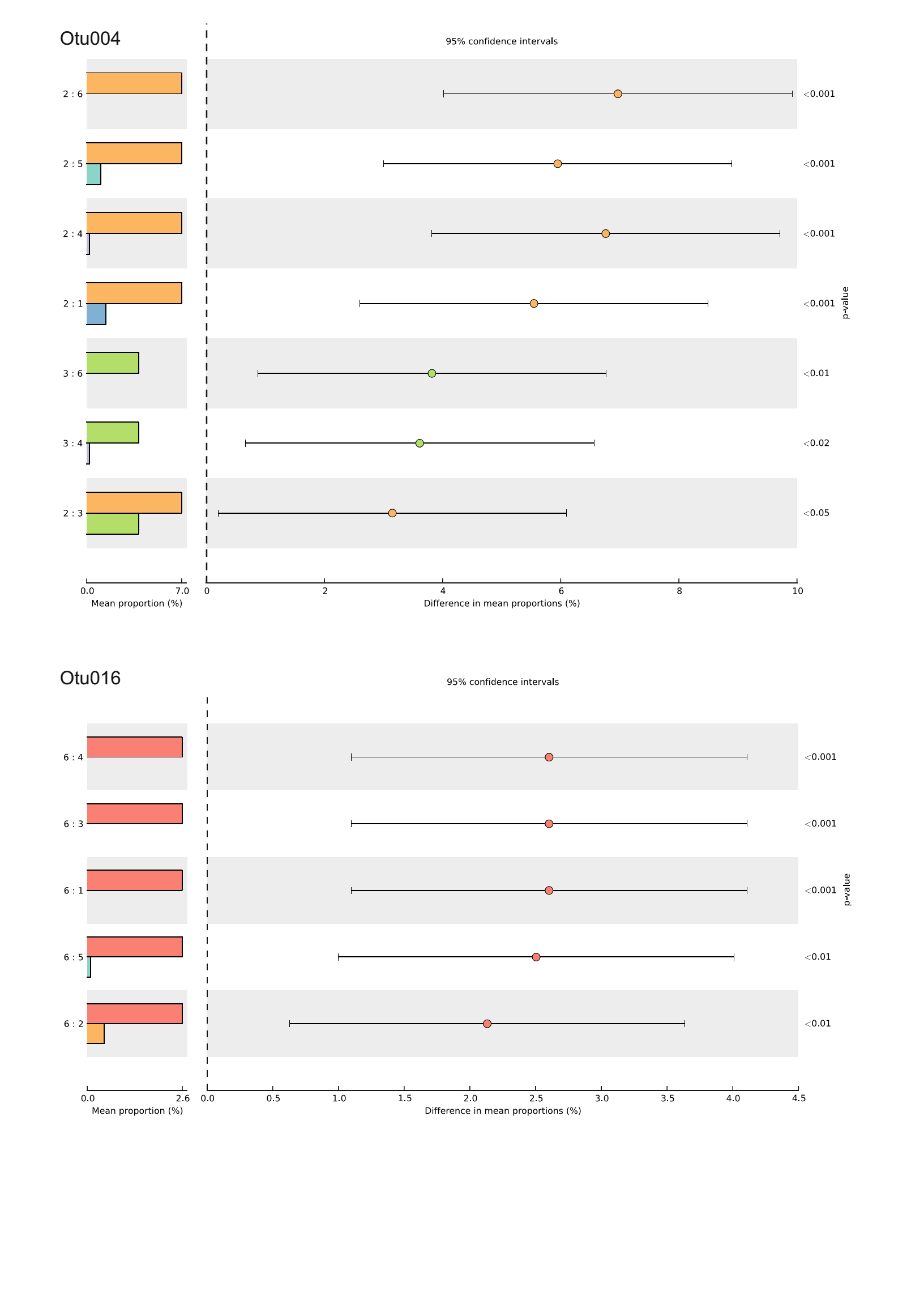


**Figure S3 continued**


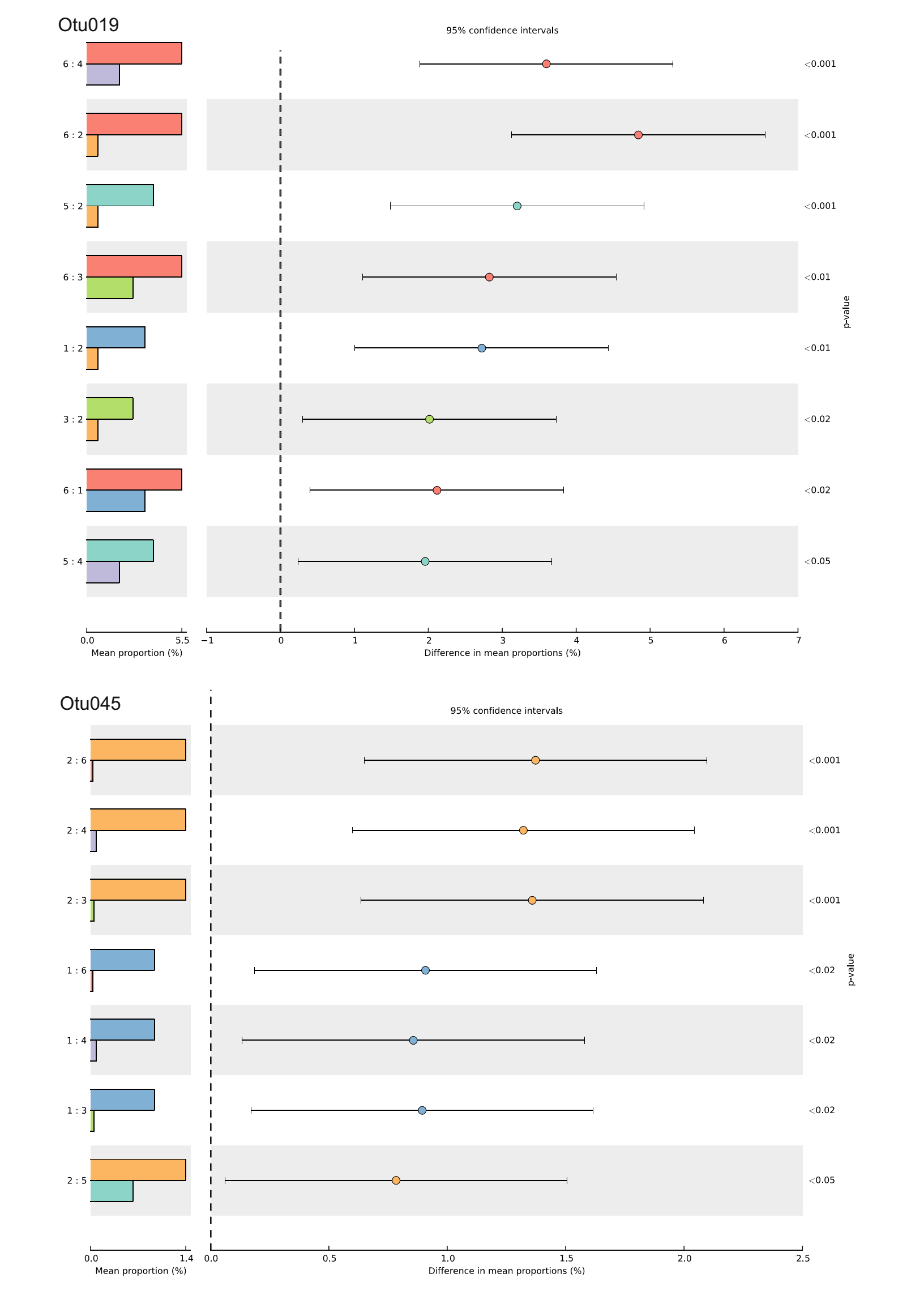


**Figure S3 continued**


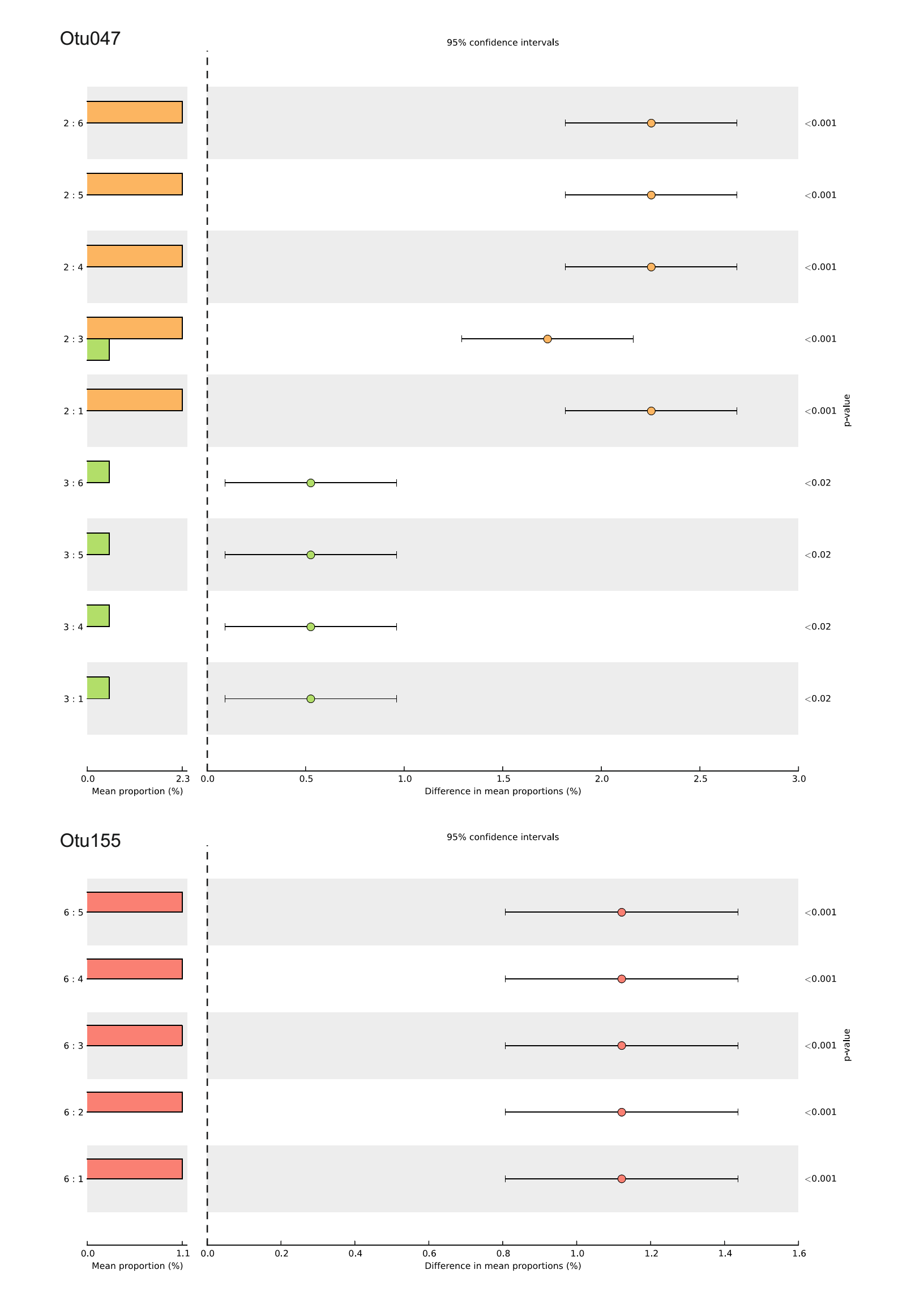


**Figure S3:** ANOVA of the means of OTUs, demonstrating that several OTUs varied between cages at each time point.
