# Supplementary material for: Age and Microenvironment Outweigh Genetic Influence on the Zucker Rat Microbiome
Source: PLoS One. 2014 Sep 18;9(9):e100916. doi: 10.1371/journal.pone.0100916 (PMC4169429; doi:10.1371/journal.pone.0100916)
Supplement: Figure S4 — PCA scores plots generated using relative abundance values of the three most abundant phyla: Bacteroidetes , Firmicutes and Actinobacteria , in samples collected from all animals at all time points (mean centred, Pareto-scaled data; R2 = 0.99, Q2 = 0.96). Principal components 1 and 2 (PC1 and PC2) are shown with the percentage of explained variance described by each component. A: Samples are coloured according to the age (in weeks) at which the sample was collected. B: Samples are coloured according to the genotype of the animal. C: Samples are coloured according to the cage (1–6) of each animal. The scores plot in (A) can be used as a reference for the sample time points; the time points are not shown in (B) and (C) to aid visualisation of potential trends. (DOCX) [file pone.0100916.s004.docx]

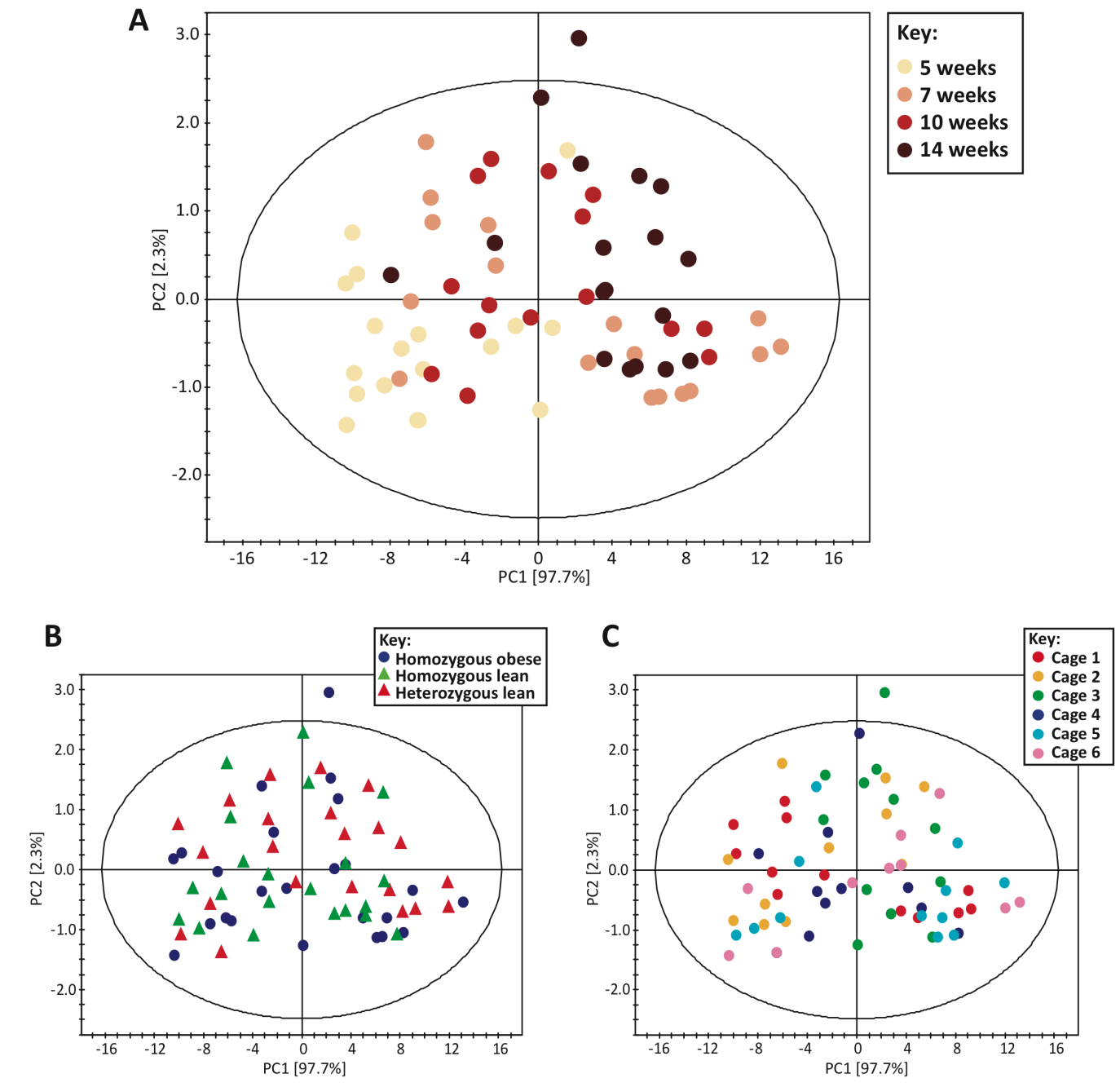


Figure S4: PCA scores plots generated using relative abundance values of the three most abundant phyla: *Bacteroidetes*, *Firmicutes* and *Actinobacteria*, in samples collected from all animals at all time points (mean centred, Pareto-scaled data; R^2^ = 0.99, Q^2^ = 0.96). Principal components 1 and 2 (PC1 and PC2) are shown with the percentage of explained variance described by each component. A: Samples are coloured according to the age (in weeks) at which the sample was collected. B: Samples are coloured according to the genotype of the animal. C: Samples are coloured according to the cage (1-6) of each animal. The scores plot in (A) can be used as a reference for the sample time points; the time points are not shown in (B) and (C) to aid visualisation of potential trends.
