# Supplementary material for: Age and Microenvironment Outweigh Genetic Influence on the Zucker Rat Microbiome
Source: PLoS One. 2014 Sep 18;9(9):e100916. doi: 10.1371/journal.pone.0100916 (PMC4169429; doi:10.1371/journal.pone.0100916)
Supplement: Figure S5 — PCA scores plots generated using relative abundance values of the six most abundant families: Bacteroidaceae, Porphyromonadaceae, Rikenellaceae, Lachnospiraceae, Ruminococcaceae and Peptostreptococcaceae , in samples collected from all animals at all time points (Log10 transformed, mean centred data; R2 = 0.83, Q2 = 0.01). Principal components 1 and 3 (PC1 and PC3) are shown with the percentage of explained variance described by each component. A: Samples are coloured according to the age (in weeks) at which the sample was collected. B: Samples are coloured according to the genotype of the animal. C: Samples are coloured according to the cage (1–6) of each animal. The scores plot in (A) can be used as a reference for the sample time points; the time points are not shown in (B) and (C) to aid visualisation of potential trends. (DOCX) [file pone.0100916.s005.docx]

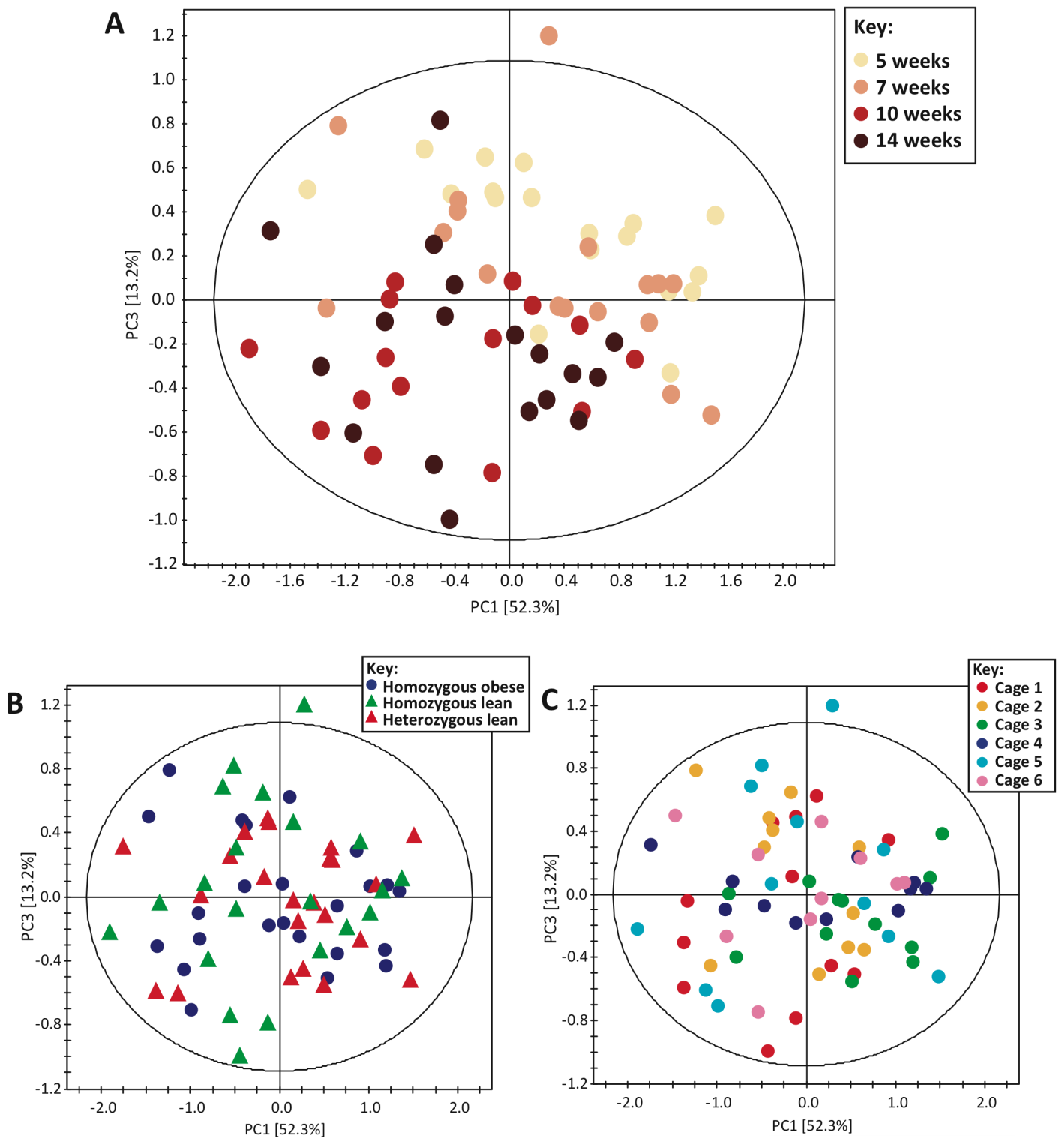


**Figure S5**: PCA scores plots generated using relative abundance values of the six most abundant families: *Bacteroidaceae, Porphyromonadaceae, Rikenellaceae, Lachnospiraceae, Ruminococcaceae* and *Peptostreptococcaceae*, in samples collected from all animals at all time points (Log_10_ transformed, mean centred data; R^2^ = 0.83, Q^2^ = 0.01). Principal components 1 and 3 (PC1 and PC3) are shown with the percentage of explained variance described by each component. A: Samples are coloured according to the age (in weeks) at which the sample was collected. B: Samples are coloured according to the genotype of the animal. C: Samples are coloured according to the cage (1-6) of each animal. The scores plot in (A) can be used as a reference for the sample time points; the time points are not shown in (B) and (C) to aid visualisation of potential trends.
