# Supplementary material for: Age and Microenvironment Outweigh Genetic Influence on the Zucker Rat Microbiome
Source: PLoS One. 2014 Sep 18;9(9):e100916. doi: 10.1371/journal.pone.0100916 (PMC4169429; doi:10.1371/journal.pone.0100916)
Supplement: Figure S6 — PCA scores plots generated using relative abundance values of the three most abundant phyla: Bacteroidetes , Firmicutes and Actinobacteria . Plots are shown for samples collected from all animals at weeks 5, 7, 10 and 14 (mean centred, Pareto-scaled data; Week 5: R2 = 1.00 Q2 = 0.92; Week 7: R2 = 1.00 Q2 = 0.98; Week 10: R2 = 1.00 Q2 = 0.97; Week 14: R2 = 1.00 Q2 = 0.95). In each plot principal components 1 and 2 (PC1 and PC2) are shown with the percentage of explained variance described by each component. Samples are coloured according to the cage (1–6) of each animal. (DOCX) [file pone.0100916.s006.docx]

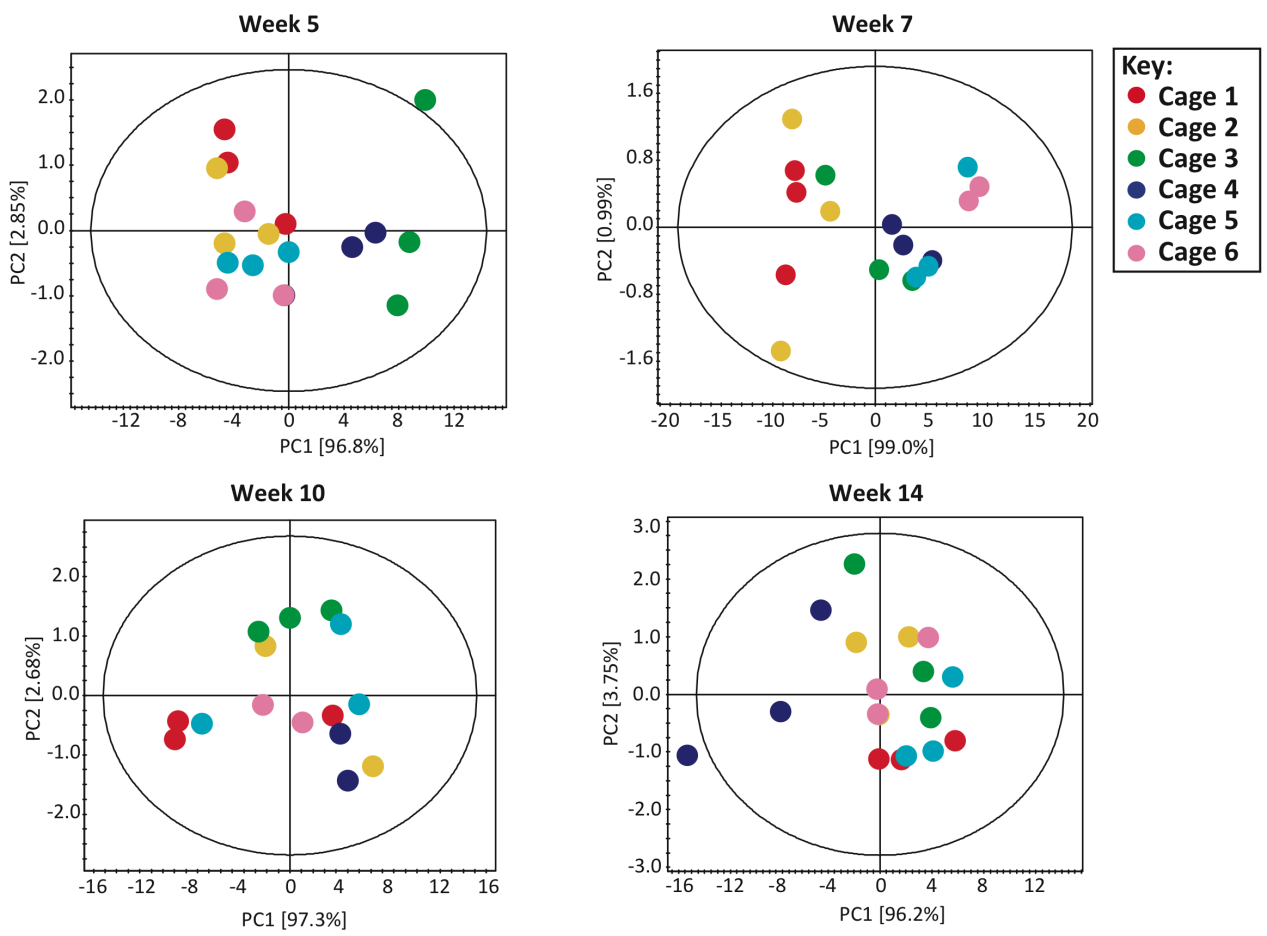


**Figure S6:** PCA scores plots generated using relative abundance values of the three most abundant phyla: *Bacteroidetes*, *Firmicutes* and *Actinobacteria*. Plots are shown for samples collected from all animals at weeks 5, 7, 10 and 14 (mean centred, Pareto-scaled data; Week 5: R^2^ = 1.00 Q^2^ = 0.92; Week 7: R^2^ = 1.00 Q^2^ = 0.98; Week 10: R^2^ = 1.00 Q^2^ = 0.97; Week 14: R^2^ = 1.00 Q^2^ = 0.95). In each plot principal components 1 and 2 (PC1 and PC2) are shown with the percentage of explained variance described by each component. Samples are coloured according to the cage (1-6) of each animal.
