# Supplementary material for: Age and Microenvironment Outweigh Genetic Influence on the Zucker Rat Microbiome
Source: PLoS One. 2014 Sep 18;9(9):e100916. doi: 10.1371/journal.pone.0100916 (PMC4169429; doi:10.1371/journal.pone.0100916)
Supplement: Figure S7 — PCA scores plots generated using relative abundance values of the six most abundant families: Bacteroidaceae, Porphyromonadaceae, Rikenellaceae, Lachnospiraceae, Ruminococcaceae and Peptostreptococcaceae . Plots are shown for samples collected from all animals at weeks 5, 7, and 10 (Log10 transformed, mean centred data; Week 5: R2 = 0.87 Q2 = 0.53; Week 7: R2 = 0.82 Q2 = 0.06; Week 10: R2 = 0.78 Q2 = 0.29). In each plot principal components 1 and 2 (PC1 and PC2) are shown with the percentage of explained variance described by each component. Samples are coloured according to the cage (1–6) of each animal. Week 14 is not shown here, as the Q2 was negative with the first component, and was thus not considered a valid model. (DOCX) [file pone.0100916.s007.docx]

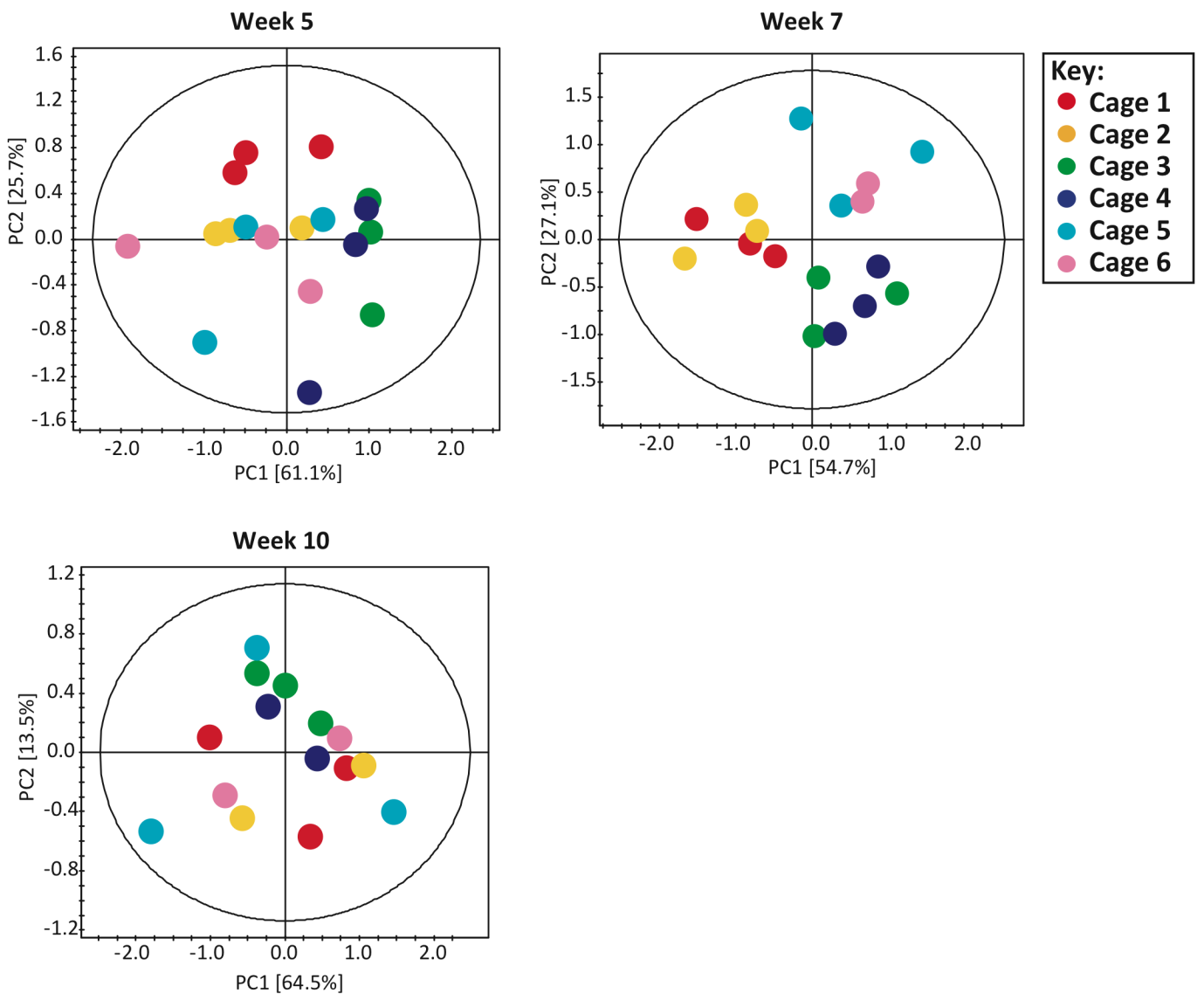


Figure S7: PCA scores plots generated using relative abundance values of the six most abundant families: *Bacteroidaceae, Porphyromonadaceae, Rikenellaceae, Lachnospiraceae, Ruminococcaceae* and *Peptostreptococcaceae*. Plots are shown for samples collected from all animals at weeks 5, 7, and 10 (Log_10_ transformed, mean centred data; Week 5: R^2^ = 0.87 Q^2^ = 0.53; Week 7: R^2^ = 0.82 Q^2^ = 0.06; Week 10: R^2^ = 0.78 Q^2^ = 0.29). In each plot principal components 1 and 2 (PC1 and PC2) are shown with the percentage of explained variance described by each component. Samples are coloured according to the cage (1-6) of each animal. Week 14 is not shown here, as the Q^2^ was negative with the first component, and was thus not considered a valid model.
