# Supplementary material for: Age and Microenvironment Outweigh Genetic Influence on the Zucker Rat Microbiome
Source: PLoS One. 2014 Sep 18;9(9):e100916. doi: 10.1371/journal.pone.0100916 (PMC4169429; doi:10.1371/journal.pone.0100916)
Supplement: Figure S8 — Box plots showing the median, lower and upper quartiles of the weighted UniFrac distances at each time point comparing the effect of genotype and cage on the community structure. Whiskers were calculated using the Tukey method; filled circles represent outliers. A lower UniFrac distance indicates greater similarity between two microbial communities (Student's t test: ns = not significant; asterisks indicate significant differences: ** P<0.01; *** P<0.001; ****P<0.0001). (DOCX) [file pone.0100916.s008.docx]

**Figure S8:** Box plots showing the median, lower and upper quartiles of the weighted UniFrac distances at each time point comparing the effect of genotype and cage on the community structure. Whiskers were calculated using the Tukey method; filled circles represent outliers. A lower UniFrac distance indicates greater similarity between two microbial communities (Student’s *t* test: ns = not significant; asterisks indicate significant differences: ** P < 0.01; *** P < 0.001; ****P < 0.0001).
