# Supplementary material for: Age and Microenvironment Outweigh Genetic Influence on the Zucker Rat Microbiome
Source: PLoS One. 2014 Sep 18;9(9):e100916. doi: 10.1371/journal.pone.0100916 (PMC4169429; doi:10.1371/journal.pone.0100916)
Supplement: Figure S10 — Relative abundances of bacteria at the family-level for all animals grouped according to cage, at each time point separately. Key: O = obese, L = homozygous lean, H = heterozygous lean. Family key: ‘Others’ composed of the families: Alcaligenaceae, Anaeroplasmataceae, Bacillaceae, Clostridiaceae, Enterobacteriaceae, Erysipelotrichaceae, Eubacteriaceae, Halomonadaceae, Incertae Sedis XIII, Incertae Sedis XIV, Lactobacillaceae, Peptococcaceae, Pseudomonadaceae and Sphingomonadaceae. (DOCX) [file pone.0100916.s010.docx]

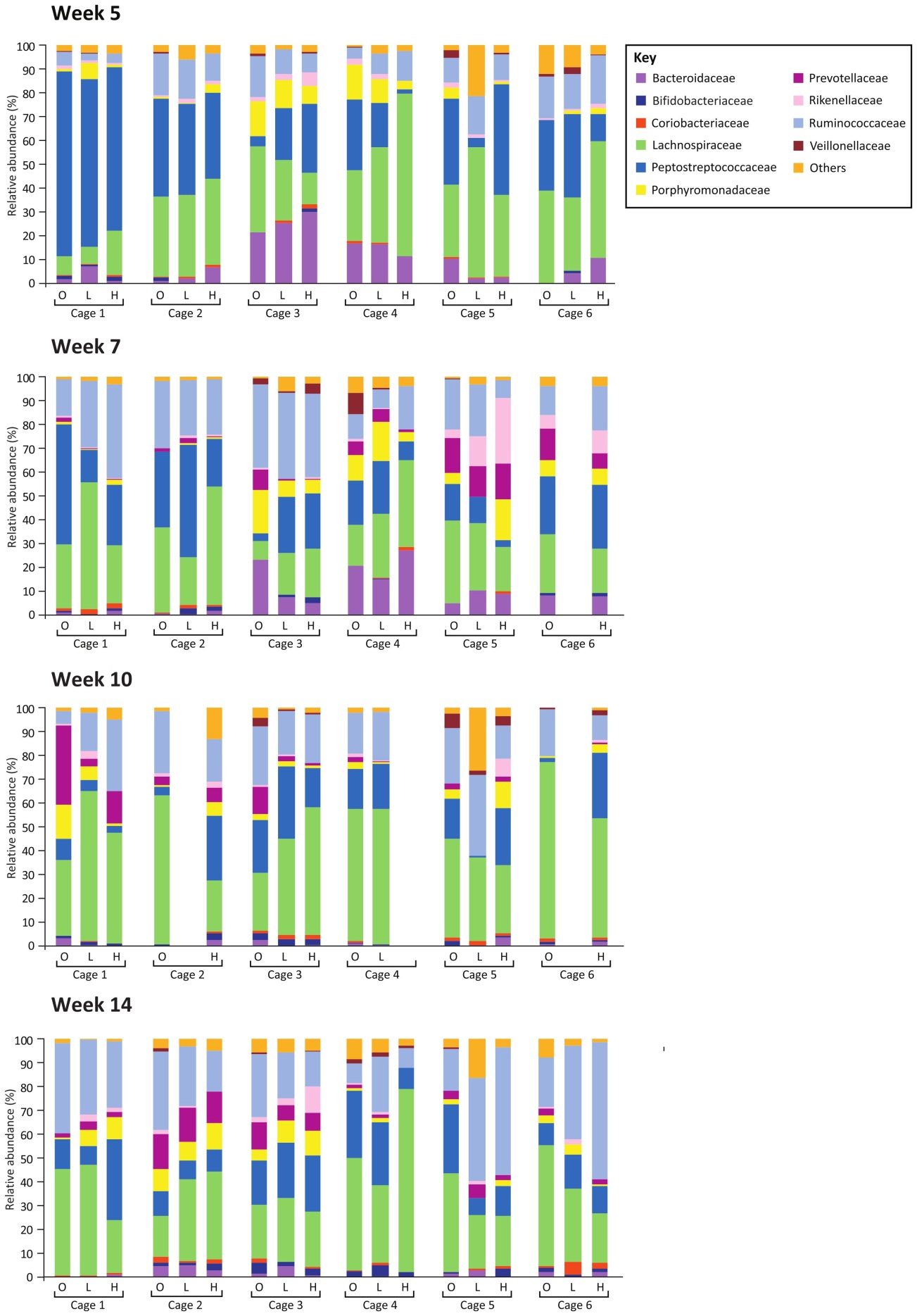


Figure S10: Relative abundances of bacteria at the family-level for all animals grouped according to cage, at each time point separately. Key: O = obese, L = homozygous lean, H = heterozygous lean. Family key: ‘Others’ composed of the families: *Alcaligenaceae, Anaeroplasmataceae, Bacillaceae, Clostridiaceae, Enterobacteriaceae, Erysipelotrichaceae, Eubacteriaceae, Halomonadaceae, Incertae Sedis XIII, Incertae Sedis XIV, Lactobacillaceae, Peptococcaceae, Pseudomonadaceae* and *Sphingomonadaceae*.
