# Supplementary material for: Age and Microenvironment Outweigh Genetic Influence on the Zucker Rat Microbiome
Source: PLoS One. 2014 Sep 18;9(9):e100916. doi: 10.1371/journal.pone.0100916 (PMC4169429; doi:10.1371/journal.pone.0100916)
Supplement: Figure S11 — ANOVA of the means of the OTU061 shows that this OTU was the only one to vary at any significant levels between cages across the 4 time points. (DOCX) [file pone.0100916.s011.docx]

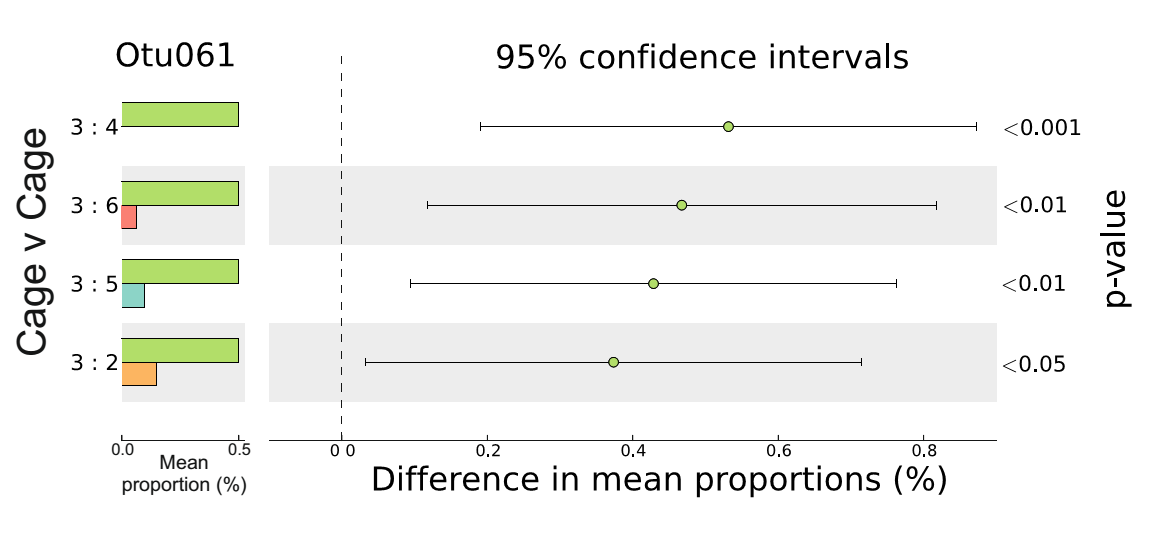


**Figure S11:** ANOVA of the means of the OTU061 shows that this OTU was the only one to vary at any significant levels between cages across the 4 time points.
