# Supplementary material for: Age and Microenvironment Outweigh Genetic Influence on the Zucker Rat Microbiome
Source: PLoS One. 2014 Sep 18;9(9):e100916. doi: 10.1371/journal.pone.0100916 (PMC4169429; doi:10.1371/journal.pone.0100916)
Supplement: Figure S12 — Body weights for each animal at 4 weeks (pre-study) and at every urine sample collection point (weeks 5 to 14). (A) obese (fa/fa) animals, (B) lean (+/+) animals and (C) lean (fa/+) animals. Colour of data points indicates cage number of the animal. (DOCX) [file pone.0100916.s012.docx]

**
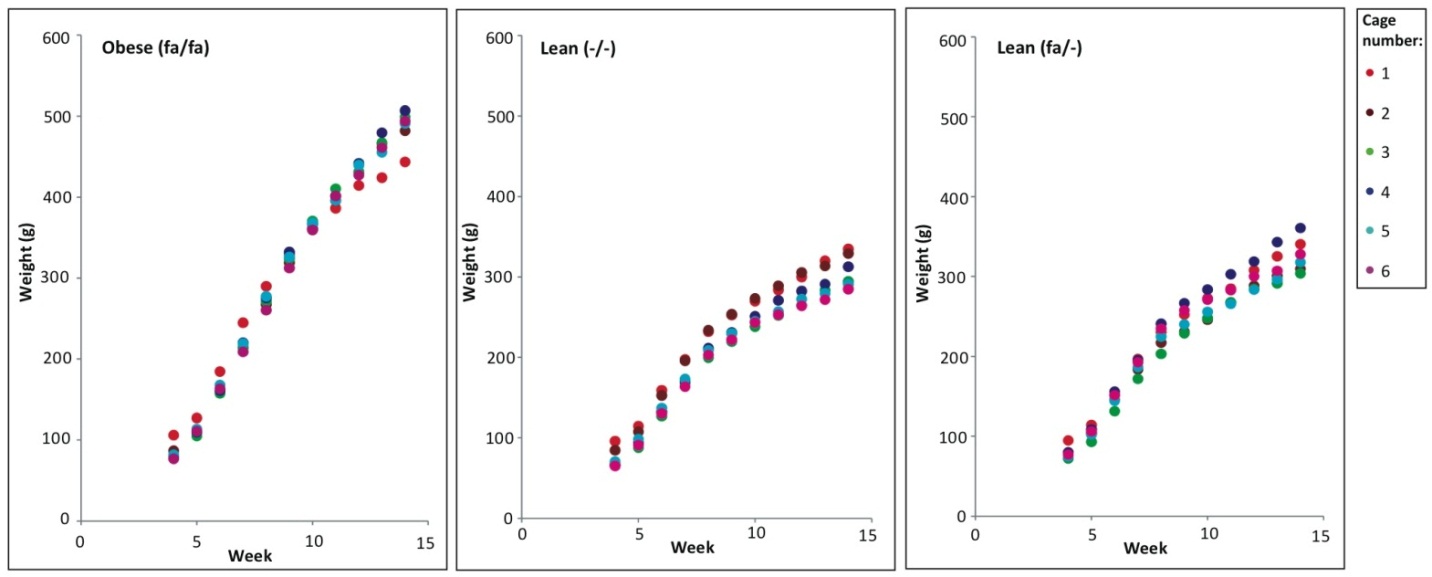
**

Figure S12: Body weights for each animal at 4 weeks (pre-study) and at every urine sample collection point (weeks 5 to 14) for: (A) obese (fa/fa) animals, (B) lean (+/+) animals and (C) lean (fa/+) animals. Colour of data points indicates cage number of the animal.
