# Supplementary material for: Age and Microenvironment Outweigh Genetic Influence on the Zucker Rat Microbiome
Source: PLoS One. 2014 Sep 18;9(9):e100916. doi: 10.1371/journal.pone.0100916 (PMC4169429; doi:10.1371/journal.pone.0100916)
Supplement: Figure S13 — Body weights for each strain at each week including pre-study (at four weeks for age), data expressed as mean ± standard error of the mean. Asterisks indicate significant differences (one-way ANOVA, followed by Tukey-Kramer multiple comparisons test, * P<0.05; ** P<0.01; *** P<0.001; **** P<0.0001). Green asterisks relate to the comparison of (fa/fa) and (+/+); red asterisks relate to the comparison of (fa/fa) and (fa/+). (DOCX) [file pone.0100916.s013.docx]

**
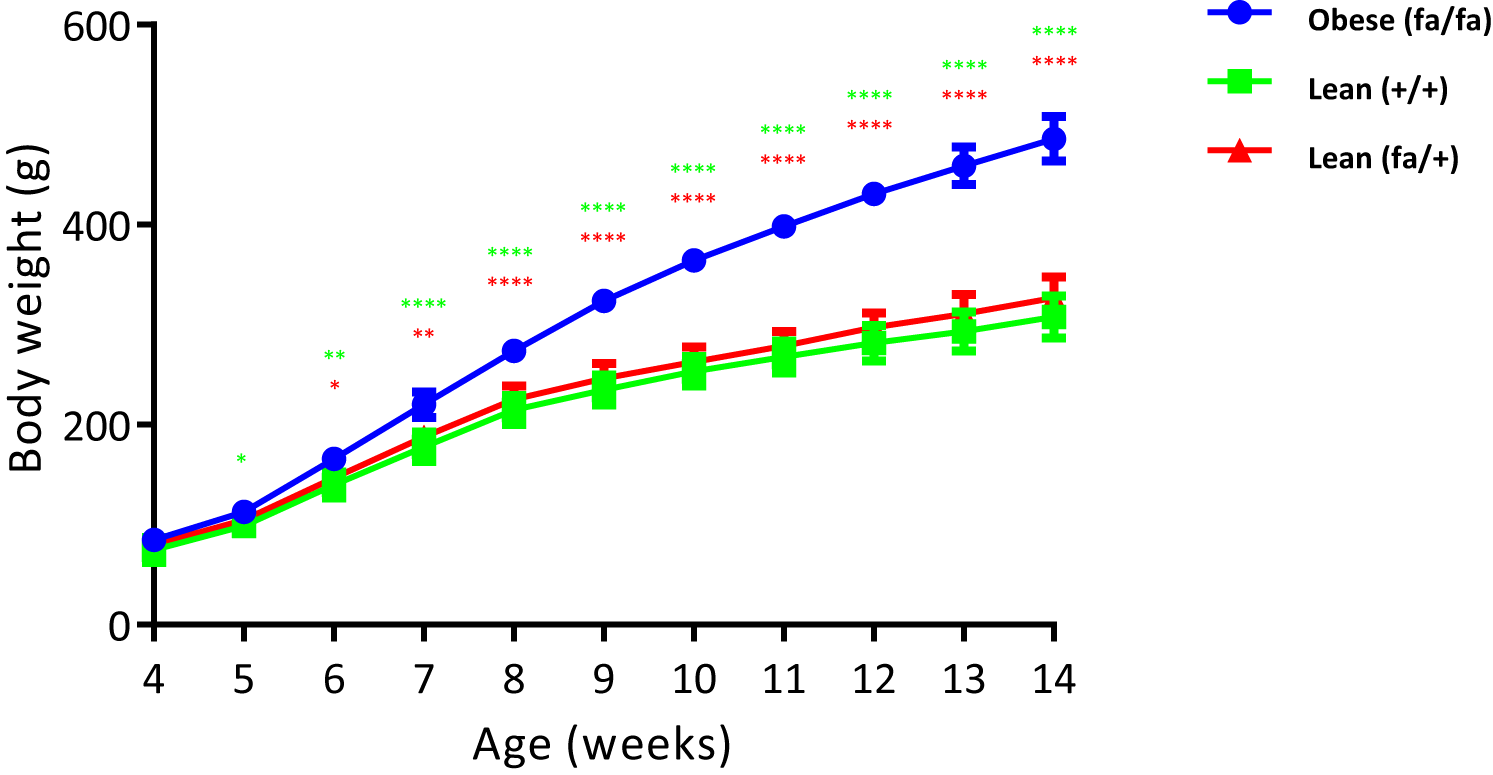
**

Figure S13: Body weights for each strain at each week including pre-study (at four weeks for age), data expressed as mean ± standard error of the mean. Asterisks indicate significant differences (one-way ANOVA, followed by Tukey-Kramer multiple comparisons test, * P < 0.05; ** P < 0.01; *** P< 0.001; **** P < 0.0001). Green asterisks relate to the comparison of (fa/fa) and (+/+); red asterisks relate to the comparison of (fa/fa) and (fa/+).
