# Supplementary material for: Age and Microenvironment Outweigh Genetic Influence on the Zucker Rat Microbiome
Source: PLoS One. 2014 Sep 18;9(9):e100916. doi: 10.1371/journal.pone.0100916 (PMC4169429; doi:10.1371/journal.pone.0100916)
Supplement: Figure S14 — Box plots of the relative abundance of Proteobacteria for each genotype at each time point. The median, lower and upper quartiles are shown. Whiskers were calculated using the Tukey method; filled circles represent outliers. Asterisks indicate significant differences (one-way ANOVA, followed by Tukey-Kramer multiple comparisons test, * P<0.05; ** P<0.01; *** P<0.001). (DOCX) [file pone.0100916.s014.docx]

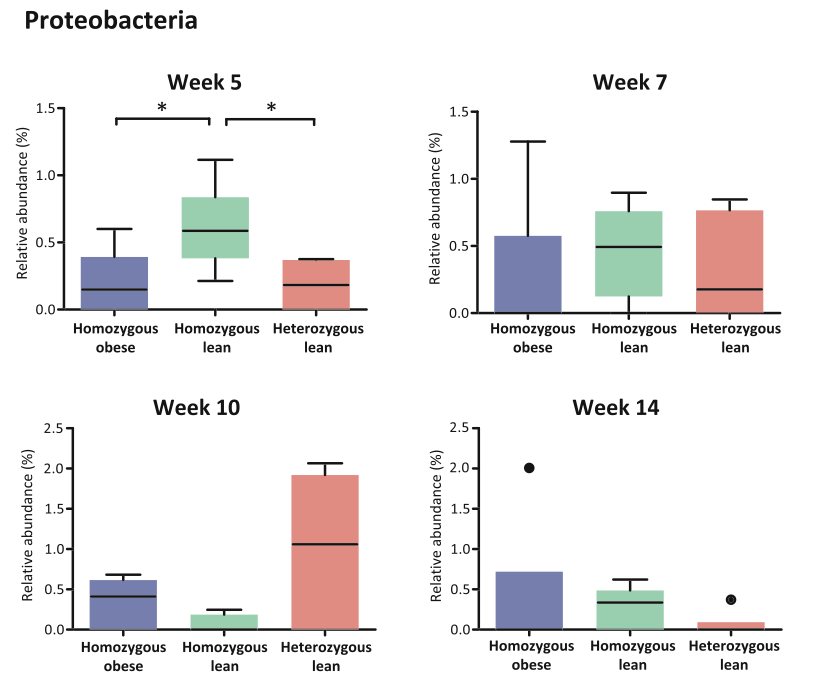


Figure S14: Box plots of the relative abundance of *Proteobacteria* for each genotype at each time point. The median, lower and upper quartiles are shown. Whiskers were calculated using the Tukey method; filled circles represent outliers. Asterisks indicate significant differences (one-way ANOVA, followed by Tukey-Kramer multiple comparisons test, * P < 0.05; ** P < 0.01; *** P< 0.001).
