# Supplementary material for: Age and Microenvironment Outweigh Genetic Influence on the Zucker Rat Microbiome
Source: PLoS One. 2014 Sep 18;9(9):e100916. doi: 10.1371/journal.pone.0100916 (PMC4169429; doi:10.1371/journal.pone.0100916)
Supplement: Figure S15 — A: mean relative abundances of each phylum for each genotype (all time points included). B: mean relative abundances of each phylum for each genotype at each time point separately. Phylum key: ‘Others’ composed of TM7 and Verrucomicrobia. (DOCX) [file pone.0100916.s015.docx]

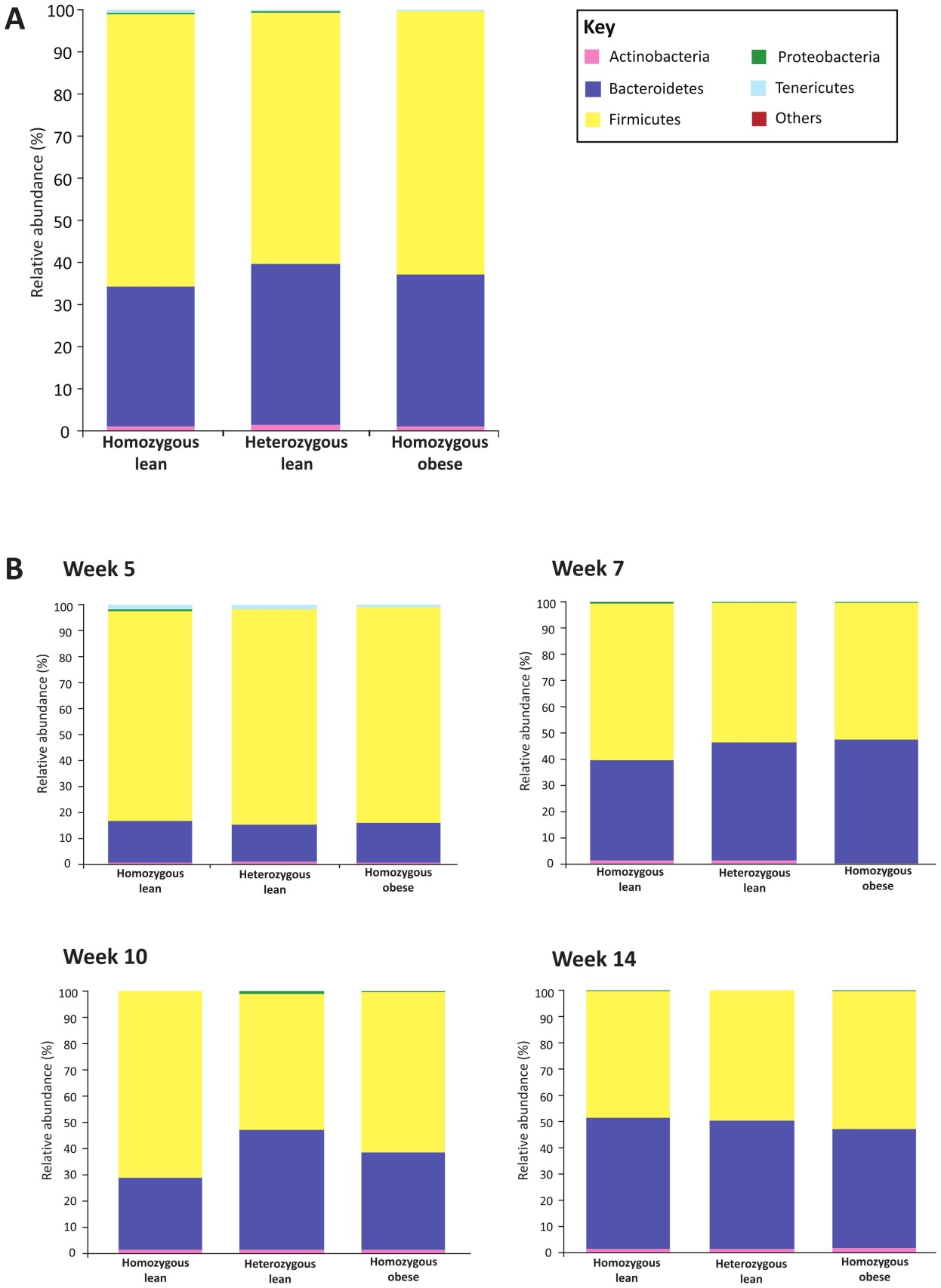


Figure S15: A: mean relative abundances of each phylum for each genotype (all time points included). B: mean relative abundances of each phylum for each genotype at each time point separately. Phylum key: ‘Others’ composed of TM7 and *Verrucomicrobia*.
