# Supplementary material for: Age and Microenvironment Outweigh Genetic Influence on the Zucker Rat Microbiome
Source: PLoS One. 2014 Sep 18;9(9):e100916. doi: 10.1371/journal.pone.0100916 (PMC4169429; doi:10.1371/journal.pone.0100916)
Supplement: Figure S16 — A: mean relative abundances of each family for each genotype (all time points included). B: mean relative abundances of each family for each genotype at each time point separately. Family key: ‘Others’ composed of the families: Alcaligenaceae, Anaeroplasmataceae, Bacillaceae, Clostridiaceae, Enterobacteriaceae, Erysipelotrichaceae, Eubacteriaceae, Halomonadaceae, Incertae Sedis XIII, Incertae Sedis XIV, Lactobacillaceae, Peptococcaceae, Pseudomonadaceae and Sphingomonadaceae. (DOCX) [file pone.0100916.s016.docx]

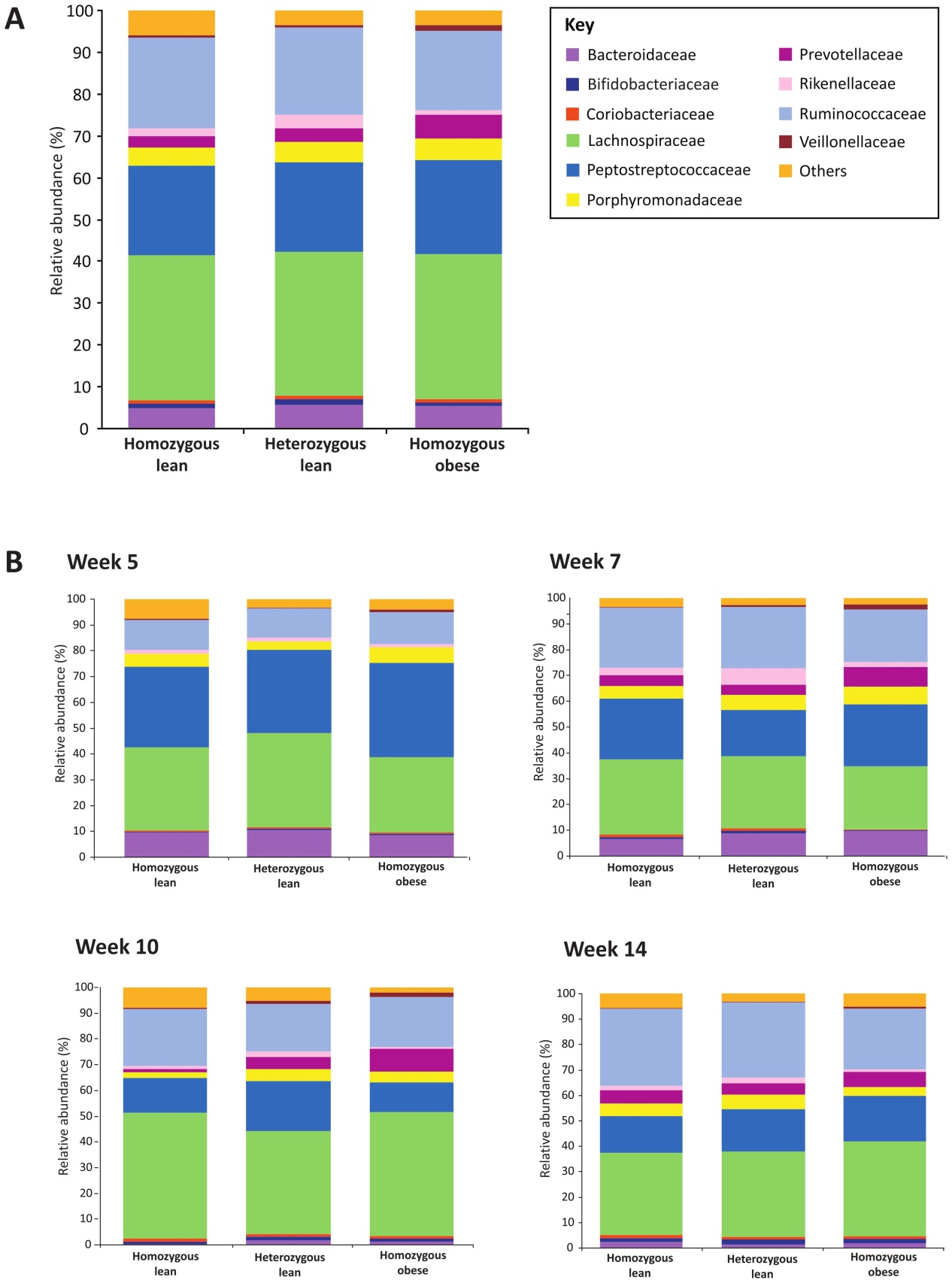


Figure S16: A: mean relative abundances of each family for each genotype (all time points included). B: mean relative abundances of each family for each genotype at each time point separately. Family key: ‘Others’ composed of the families: *Alcaligenaceae, Anaeroplasmataceae, Bacillaceae, Clostridiaceae, Enterobacteriaceae, Erysipelotrichaceae, Eubacteriaceae, Halomonadaceae, Incertae Sedis XIII, Incertae Sedis XIV, Lactobacillaceae, Peptococcaceae, Pseudomonadaceae* and *Sphingomonadaceae*.
