# Supplementary material for: Age and Microenvironment Outweigh Genetic Influence on the Zucker Rat Microbiome
Source: PLoS One. 2014 Sep 18;9(9):e100916. doi: 10.1371/journal.pone.0100916 (PMC4169429; doi:10.1371/journal.pone.0100916)
Supplement: Table S1 — Primers used to amplify the V1-V3 regions of the 16S rRNA gene. The unique barcode for each sample is shown in red, and allowed for multiplexing of the samples on the 454 sequencer on three different PTPs (Pico Titre Plate 8ths, 1 2 or 3). (DOCX) [file pone.0100916.s017.docx]

| Forward primer | | | | | 5' GCCTAACACATGCAAGTC 3' |
| --- | --- | --- | --- | --- | --- |
| Reverse primer | | | | | 5' ATTACCGCGGCTGCTGG 3' |
|  | | | | | |
| Sample | Animal | Genotype | Week | Plate | Full sequences including adapter and barcode (unique to each plate) |
| **1** | 1 | Obese | 5 | **1** | CCATCTCATCCCTGCGTGTCTCCGACTCAG GATCT GCCTAACACATGCAAGTC |
| **2** | 2 | Hom lean | 5 |  | CCATCTCATCCCTGCGTGTCTCCGACTCAG ATCAG GCCTAACACATGCAAGTC |
| **3** | 3 | Het lean | 5 |  | CCATCTCATCCCTGCGTGTCTCCGACTCAG ACACT GCCTAACACATGCAAGTC |
| **4** | 4 | Obese | 5 |  | CCATCTCATCCCTGCGTGTCTCCGACTCAG AGCTA GCCTAACACATGCAAGTC |
| **5** | 5 | Hom lean | 5 |  | CCATCTCATCCCTGCGTGTCTCCGACTCAG CACAC GCCTAACACATGCAAGTC |
| **6** | 6 | Het lean | 5 |  | CCATCTCATCCCTGCGTGTCTCCGACTCAG ACAGA GCCTAACACATGCAAGTC |
| **7** | 7 | Obese | 5 |  | CCATCTCATCCCTGCGTGTCTCCGACTCAG AGATG GCCTAACACATGCAAGTC |
| **8** | 8 | Hom lean | 5 |  | CCATCTCATCCCTGCGTGTCTCCGACTCAG CACTG GCCTAACACATGCAAGTC |
| **9** | 9 | Het lean | 5 |  | CCATCTCATCCCTGCGTGTCTCCGACTCAG CAGAG GCCTAACACATGCAAGTC |
| **10** | 10 | Obese | 5 |  | CCATCTCATCCCTGCGTGTCTCCGACTCAG CGCAG GCCTAACACATGCAAGTC |
| **11** | 11 | Hom lean | 5 |  | CCATCTCATCCCTGCGTGTCTCCGACTCAG CTGTG GCCTAACACATGCAAGTC |
| **12** | 12 | Het lean | 5 |  | CCATCTCATCCCTGCGTGTCTCCGACTCAG GTGAG GCCTAACACATGCAAGTC |
| **13** | 13 | Obese | 5 |  | CCATCTCATCCCTGCGTGTCTCCGACTCAG TCATG GCCTAACACATGCAAGTC |
| **14** | 14 | Hom lean | 5 |  | CCATCTCATCCCTGCGTGTCTCCGACTCAG AGCAT GCCTAACACATGCAAGTC |
| **15** | 15 | Het lean | 5 |  | CCATCTCATCCCTGCGTGTCTCCGACTCAG CAGCT GCCTAACACATGCAAGTC |
| **16** | 16 | Obese | 5 |  | CCATCTCATCCCTGCGTGTCTCCGACTCAG CATGT GCCTAACACATGCAAGTC |
| **17** | 17 | Hom lean | 5 |  | CCATCTCATCCCTGCGTGTCTCCGACTCAG CTGAT GCCTAACACATGCAAGTC |
| **18** | 18 | Het lean | 5 |  | CCATCTCATCCCTGCGTGTCTCCGACTCAG CTGCA GCCTAACACATGCAAGTC |
| **19** | 1 | Obese | 7 |  | CCATCTCATCCCTGCGTGTCTCCGACTCAG GATGA GCCTAACACATGCAAGTC |
| **20** | 2 | Hom lean | 7 |  | CCATCTCATCCCTGCGTGTCTCCGACTCAG TACGC GCCTAACACATGCAAGTC |
| **21** | 3 | Het lean | 7 |  | CCATCTCATCCCTGCGTGTCTCCGACTCAG ACTGC GCCTAACACATGCAAGTC |
| **22** | 4 | Obese | 7 |  | CCATCTCATCCCTGCGTGTCTCCGACTCAG GTCAC GCCTAACACATGCAAGTC |
| **23** | 5 | Hom lean | 7 |  | CCATCTCATCCCTGCGTGTCTCCGACTCAG CGTAC GCCTAACACATGCAAGTC |

| Sample | Animal | Genotype | Week | Plate | Full sequences including adapter and barcode (unique to each plate) |
| --- | --- | --- | --- | --- | --- |
| **24** | 6 | Het lean | 7 | **2** | CCATCTCATCCCTGCGTGTCTCCGACTCAG GATCT GCCTAACACATGCAAGTC |
| **25** | 7 | Obese | 7 |  | CCATCTCATCCCTGCGTGTCTCCGACTCAG ATCAG GCCTAACACATGCAAGTC |
| **26** | 8 | Hom lean | 7 |  | CCATCTCATCCCTGCGTGTCTCCGACTCAG ACACT GCCTAACACATGCAAGTC |
| **27** | 9 | Het lean | 7 |  | CCATCTCATCCCTGCGTGTCTCCGACTCAG AGCTA GCCTAACACATGCAAGTC |
| **28** | 10 | Obese | 7 |  | CCATCTCATCCCTGCGTGTCTCCGACTCAG CACAC GCCTAACACATGCAAGTC |
| **29** | 11 | Hom lean | 7 |  | CCATCTCATCCCTGCGTGTCTCCGACTCAG ACAGA GCCTAACACATGCAAGTC |
| **30** | 12 | Het lean | 7 |  | CCATCTCATCCCTGCGTGTCTCCGACTCAG AGATG GCCTAACACATGCAAGTC |
| **31** | 13 | Obese | 7 |  | CCATCTCATCCCTGCGTGTCTCCGACTCAG CACTG GCCTAACACATGCAAGTC |
| **32** | 14 | Hom lean | 7 |  | CCATCTCATCCCTGCGTGTCTCCGACTCAG CAGAG GCCTAACACATGCAAGTC |
| **33** | 15 | Het lean | 7 |  | CCATCTCATCCCTGCGTGTCTCCGACTCAG CGCAG GCCTAACACATGCAAGTC |
| **34** | 16 | Obese | 7 |  | CCATCTCATCCCTGCGTGTCTCCGACTCAG CTGTG GCCTAACACATGCAAGTC |
| **35** | 18 | Het lean | 7 |  | CCATCTCATCCCTGCGTGTCTCCGACTCAG GTGAG GCCTAACACATGCAAGTC |
| **36** | 1 | Obese | 10 |  | CCATCTCATCCCTGCGTGTCTCCGACTCAG TCATG GCCTAACACATGCAAGTC |
| **37** | 2 | Hom lean | 10 |  | CCATCTCATCCCTGCGTGTCTCCGACTCAG AGCAT GCCTAACACATGCAAGTC |
| **38** | 3 | Het lean | 10 |  | CCATCTCATCCCTGCGTGTCTCCGACTCAG CAGCT GCCTAACACATGCAAGTC |
| **39** | 4 | Obese | 10 |  | CCATCTCATCCCTGCGTGTCTCCGACTCAG CATGT GCCTAACACATGCAAGTC |
| **40** | 6 | Het lean | 10 |  | CCATCTCATCCCTGCGTGTCTCCGACTCAG CTGAT GCCTAACACATGCAAGTC |
| **41** | 7 | Obese | 10 |  | CCATCTCATCCCTGCGTGTCTCCGACTCAG CTGCA GCCTAACACATGCAAGTC |
| **42** | 8 | Hom lean | 10 |  | CCATCTCATCCCTGCGTGTCTCCGACTCAG GATGA GCCTAACACATGCAAGTC |
| **43** | 9 | Het lean | 10 |  | CCATCTCATCCCTGCGTGTCTCCGACTCAG TACGC GCCTAACACATGCAAGTC |
| **44** | 10 | Obese | 10 |  | CCATCTCATCCCTGCGTGTCTCCGACTCAG ACTGC GCCTAACACATGCAAGTC |
| **45** | 11 | Hom lean | 10 |  | CCATCTCATCCCTGCGTGTCTCCGACTCAG GTCAC GCCTAACACATGCAAGTC |
| **46** | 13 | Obese | 10 |  | CCATCTCATCCCTGCGTGTCTCCGACTCAG CGTAC GCCTAACACATGCAAGTC |

| Sample | Animal | Genotype | Week | Plate | Full sequences including adapter and barcode (unique to each plate) |
| --- | --- | --- | --- | --- | --- |
| **47** | 14 | Hom lean | 10 | **3** | CCATCTCATCCCTGCGTGTCTCCGACTCAG GATCT GCCTAACACATGCAAGTC |
| **48** | 15 | Het lean | 10 |  | CCATCTCATCCCTGCGTGTCTCCGACTCAG ATCAG GCCTAACACATGCAAGTC |
| **49** | 16 | Obese | 10 |  | CCATCTCATCCCTGCGTGTCTCCGACTCAG ACACT GCCTAACACATGCAAGTC |
| **50** | 18 | Het lean | 10 |  | CCATCTCATCCCTGCGTGTCTCCGACTCAG AGCTA GCCTAACACATGCAAGTC |
| **51** | 1 | Obese | 14 |  | CCATCTCATCCCTGCGTGTCTCCGACTCAG CACAC GCCTAACACATGCAAGTC |
| **52** | 2 | Hom lean | 14 |  | CCATCTCATCCCTGCGTGTCTCCGACTCAG ACAGA GCCTAACACATGCAAGTC |
| **53** | 3 | Het lean | 14 |  | CCATCTCATCCCTGCGTGTCTCCGACTCAG AGATG GCCTAACACATGCAAGTC |
| **54** | 4 | Obese | 14 |  | CCATCTCATCCCTGCGTGTCTCCGACTCAG CACTG GCCTAACACATGCAAGTC |
| **55** | 5 | Hom lean | 14 |  | CCATCTCATCCCTGCGTGTCTCCGACTCAG CAGAG GCCTAACACATGCAAGTC |
| **56** | 6 | Het lean | 14 |  | CCATCTCATCCCTGCGTGTCTCCGACTCAG CGCAG GCCTAACACATGCAAGTC |
| **57** | 7 | Obese | 14 |  | CCATCTCATCCCTGCGTGTCTCCGACTCAG CTGTG GCCTAACACATGCAAGTC |
| **58** | 8 | Hom lean | 14 |  | CCATCTCATCCCTGCGTGTCTCCGACTCAG GTGAG GCCTAACACATGCAAGTC |
| **59** | 9 | Het lean | 14 |  | CCATCTCATCCCTGCGTGTCTCCGACTCAG TCATG GCCTAACACATGCAAGTC |
| **60** | 10 | Obese | 14 |  | CCATCTCATCCCTGCGTGTCTCCGACTCAG AGCAT GCCTAACACATGCAAGTC |
| **61** | 11 | Hom lean | 14 |  | CCATCTCATCCCTGCGTGTCTCCGACTCAG CAGCT GCCTAACACATGCAAGTC |
| **62** | 12 | Het lean | 14 |  | CCATCTCATCCCTGCGTGTCTCCGACTCAG CATGT GCCTAACACATGCAAGTC |
| **63** | 13 | Obese | 14 |  | CCATCTCATCCCTGCGTGTCTCCGACTCAG CTGAT GCCTAACACATGCAAGTC |
| **64** | 14 | Hom lean | 14 |  | CCATCTCATCCCTGCGTGTCTCCGACTCAG CTGCA GCCTAACACATGCAAGTC |
| **65** | 15 | Het lean | 14 |  | CCATCTCATCCCTGCGTGTCTCCGACTCAG GATGA GCCTAACACATGCAAGTC |
| **66** | 16 | Obese | 14 |  | CCATCTCATCCCTGCGTGTCTCCGACTCAG TACGC GCCTAACACATGCAAGTC |
| **67** | 17 | Hom lean | 14 |  | CCATCTCATCCCTGCGTGTCTCCGACTCAG ACTGC GCCTAACACATGCAAGTC |
| **68** | 18 | Het lean | 14 |  | CCATCTCATCCCTGCGTGTCTCCGACTCAG GTCAC GCCTAACACATGCAAGTC |
| Reverse primer | | | | | CCTATCCCCTGTGTGCCTTGGCAGTCTCAG ATTACCGCGGCTGCTGG |
