# Supplementary material for: Age and Microenvironment Outweigh Genetic Influence on the Zucker Rat Microbiome
Source: PLoS One. 2014 Sep 18;9(9):e100916. doi: 10.1371/journal.pone.0100916 (PMC4169429; doi:10.1371/journal.pone.0100916)
Supplement: Table S2 — Sequence counts per sample. (DOCX) [file pone.0100916.s018.docx]

**Table S2**: Sequence counts per sample.

| **Animal** | **Tag** | **animal no** | **Genotype** | **Week** | **Sequences after trimming** |
| --- | --- | --- | --- | --- | --- |
| **1** | 1_1_HL1 | 1 | Obese | 5 | 695 |
|  | 1_2_HL1 | 1 | Obese | 7 | 585 |
|  | 1_3_HL2 | 1 | Obese | 10 | 294 |
|  | 1_4_HL3 | 1 | Obese | 14 | 535 |
| **2** | 2_1_HL1 | 2 | Hom lean | 5 | 545 |
|  | 2_2_HL1 | 2 | Hom lean | 7 | 463 |
|  | 2_3_HL2 | 2 | Hom lean | 10 | 411 |
|  | 2_4_HL3 | 2 | Hom lean | 14 | 1227 |
| **3** | 3_1_HL1 | 3 | Het lean | 5 | 867 |
|  | 3_2_HL1 | 3 | Het lean | 7 | 741 |
|  | 3_3_HL2 | 3 | Het lean | 10 | 282 |
|  | 3_4_HL3 | 3 | Het lean | 14 | 975 |
| **4** | 4_1_HL1 | 4 | Obese | 5 | 413 |
|  | 4_2_HL1 | 4 | Obese | 7 | 732 |
|  | 4_3_HL2 | 4 | Obese | 10 | 188 |
|  | 4_4_HL3 | 4 | Obese | 14 | 637 |
| **5** | 5_1_HL1 | 5 | Hom lean | 5 | 483 |
|  | 5_2_HL1 | 5 | Hom lean | 7 | 860 |
|  | 5_4_HL3 | 5 | Hom lean | 14 | 744 |
| **6** | 6_1_HL1 | 6 | Het lean | 5 | 609 |
|  | 6_2_HL2 | 6 | Het lean | 7 | 344 |
|  | 6_3_HL2 | 6 | Het lean | 10 | 246 |
|  | 6_4_HL3 | 6 | Het lean | 14 | 648 |
| **7** | 7_1_HL1 | 7 | Obese | 5 | 533 |
|  | 7_2_HL2 | 7 | Obese | 7 | 344 |
|  | 7_3_HL2 | 7 | Obese | 10 | 407 |
|  | 7_4_HL3 | 7 | Obese | 14 | 1005 |
| **8** | 8_1_HL1 | 8 | Hom lean | 5 | 417 |
|  | 8_2_HL2 | 8 | Hom lean | 7 | 237 |
|  | 8_3_HL2 | 8 | Hom lean | 10 | 227 |
|  | 8_4_HL3 | 8 | Hom lean | 14 | 840 |
| **9** | 9_1_HL1 | 9 | Het lean | 5 | 434 |
|  | 9_2_HL2 | 9 | Het lean | 7 | 284 |
|  | 9_3_HL2 | 9 | Het lean | 10 | 416 |
|  | 9_4_HL3 | 9 | Het lean | 14 | 705 |
| **10** | 10_1_HL1 | 10 | Obese | 5 | 479 |
|  | 10_2_HL2 | 10 | Obese | 7 | 366 |
|  | 10_3_HL2 | 10 | Obese | 10 | 314 |
|  | 10_4_HL3 | 10 | Obese | 14 | 583 |
| **11** | 11_1_HL1 | 11 | Hom lean | 5 | 425 |
|  | 11_2_HL2 | 11 | Hom lean | 7 | 412 |
|  | 11_3_HL2 | 11 | Hom lean | 10 | 415 |
|  | 11_4_HL3 | 11 | Hom lean | 14 | 404 |
| **12** | 12_1_HL1 | 12 | Het lean | 5 | 423 |
|  | 12_2_HL2 | 12 | Het lean | 7 | 119 |
|  | 12_4_HL3 | 12 | Het lean | 14 | 369 |
| **13** | 13_1_HL1 | 13 | Obese | 5 | 338 |
|  | 13_2_HL2 | 13 | Obese | 7 | 402 |
|  | 13_3_HL2 | 13 | Obese | 10 | 440 |
|  | 13_4_HL3 | 13 | Obese | 14 | 823 |
| **14** | 14_1_HL1 | 14 | Hom lean | 5 | 323 |
|  | 14_2_HL2 | 14 | Hom lean | 7 | 272 |
|  | 14_3_HL3 | 14 | Hom lean | 10 | 476 |
|  | 14_4_HL3 | 14 | Hom lean | 14 | 737 |
| **15** | 15_1_HL1 | 15 | Het lean | 5 | 414 |
|  | 15_2_HL2 | 15 | Het lean | 7 | 271 |
|  | 15_3_HL3 | 15 | Het lean | 10 | 885 |
|  | 15_4_HL3 | 15 | Het lean | 14 | 1042 |
| **16** | 16_1_HL1 | 16 | Obese | 5 | 292 |
|  | 16_2_HL2 | 16 | Obese | 7 | 249 |
|  | 16_3_HL3 | 16 | Obese | 10 | 1048 |
|  | 16_4_HL3 | 16 | Obese | 14 | 936 |
| **17** | 17_1_HL1 | 17 | Hom lean | 5 | 472 |
|  | 17_4_HL3 | 17 | Hom lean | 14 | 960 |
| **18** | 18_1_HL1 | 18 | Het lean | 5 | 289 |
|  | 18_2_HL2 | 18 | Het lean | 7 | 405 |
|  | 18_3_HL3 | 18 | Het lean | 10 | 1094 |
|  | 18_4_HL3 | 18 | Het lean | 14 | 569 |
