# Supplementary material for: Age and Microenvironment Outweigh Genetic Influence on the Zucker Rat Microbiome
Source: PLoS One. 2014 Sep 18;9(9):e100916. doi: 10.1371/journal.pone.0100916 (PMC4169429; doi:10.1371/journal.pone.0100916)
Supplement: Table S3 — The OTUs identified by STAMP to be significantly altered in the faecal samples when grouped by week. All the means for each group were compared using an ANOVA and multiple testing using the Bonferroni correction (see Figure S2 for more detail). (DOCX) [file pone.0100916.s019.docx]

**Table S3:**  The OTUs identified by STAMP to be significantly altered in the faecal samples when grouped by week (see supplementary Figure S2 for more detail), all the means for each group were compared using an ANOVA and multiple testing using the Bonferroni correction.

| Phylum | Genus | Feature | p-value | Corrected p-value |
| --- | --- | --- | --- | --- |
| *Firmicutes* | *Clostridium XI* | Otu001 | 9.50E-07 | 1.26E-04 |
| *Bacteroidetes* | *Meniscus* | Otu002 | 4.31E-05 | 5.33E-03 |
| *Bacteroidetes* | *Meniscus* | Otu003 | 5.35E-09 | 6.85E-09 |
| *Firmicutes* | *Papillibacter* | Otu006 | 8.36E-09 | 1.11E-06 |
| *Firmicutes* | *Syntrophococcus* | Otu007 | 1.34E-04 | 1.78E-04 |
| *Firmicutes* | *Syntrophococcus* | Otu008 | 9.78E-07 | 1.30E-02 |
| *Bacteroidetes* | *Bacteroides* | Otu017 | 7.03E-06 | 9.35E-02 |
| *Bacteroidetes* | *Meniscus* | Otu019 | 1.72E-12 | 2.30E-10 |
| *Bacteroidetes* | *Bacteroides* | Otu033 | 2.60E-04 | 0.035 |
| *Firmicutes* | *Clostridium IV* | Otu035 | 2.45E-04 | 0.033 |
| *Firmicutes* | *Marvinbryantia* | Otu036 | 7.60E-13 | 1.02E-10 |
| *Tenericutes* | *Anaeroplasma* | Otu043 | 3.33E-04 | 4.43E-02 |
| *Firmicutes* | *Lachnospiracea_incertae_sedis* | Otu046 | 3.27E-02 | 0.043 |
| *Firmicutes* | *Clostridium sensu stricto* | Otu049 | 5.98E-05 | 7.96E-03 |
| *Firmicutes* | *Acetivibrio* | Otu051 | 2.35E-03 | 3.13E-01 |
| *Firmicutes* | *Butyrivibrio* | Otu055 | 1.94E-03 | 2.58E-01 |
| *Firmicutes* | *Clostridium XlVa* | Otu063 | 9.12E-06 | 1.31E-01 |
| *Bacteroidetes* | *Bacteroides* | Otu065 | 2.37E-03 | 3.48E-01 |
| *Firmicutes* | *Marvinbryantia* | Otu073 | 2.34E-04 | 0.031 |
| *Firmicutes* | *Butyricicoccus* | Otu081 | 1.48E-03 | 1.97E-01 |
| *Firmicutes* | *Marvinbryantia* | Otu085 | 2.72E-05 | 3.42E-01 |
| *Bacteroidetes* | *Paludibacter* | Otu093 | 1.03E-04 | 0.014 |
| *Firmicutes* | *Coprococcus* | Otu177 | 1.18E-06 | 1.56E-04 |
| *Bacteroidetes* | *Alistipes* | Otu225 | 1.06E-06 | 1.41E-02 |
