# Supplementary material for: Age and Microenvironment Outweigh Genetic Influence on the Zucker Rat Microbiome
Source: PLoS One. 2014 Sep 18;9(9):e100916. doi: 10.1371/journal.pone.0100916 (PMC4169429; doi:10.1371/journal.pone.0100916)
Supplement: Table S4 — OTUs which were significantly changed at each time point between cages (P<0.05, corrected for multiple testing), see Figure S3 for more detail. (DOCX) [file pone.0100916.s020.docx]

**Table S4:** OTUs which were significantly changed at each time point between cages (P < 0.05, corrected for multiple testing), see Figure S3 for more detail.

| Time point | OTU | Genus (Family) | Range^a^ | Cage difference |
| --- | --- | --- | --- | --- |
| Week 5 | 001 | *Clostridium* *XI* (*Peptostreptococcaceae*) | 30-55% | 1 > All^b^ |
|  | 017 | *Bacteroides* | 10-15% | 3 > All |
|  | 032 | *Subdoligranulum* | 5-6% | 3 > All |
| Week 7 | 002 | *Meniscus* | 20-50% | 4 >3  5 > 3  6 > 1,2,3,4 & 5 |
|  | 009 | *Hallella* | 6-10% | 5 > All |
|  | 026 | *Marvinbryantia* | 5-6% | 2 > All |
|  | 104 | *Clostridium IV* (*Ruminococcaceae*) | 1-2% | 1 > 2,3,4 & 5  6 > 3,4 & 5 |
|  | 197 | *Meniscus* | 2.5-3% | 6 > All |
| Week 10 | 019 | *Meniscus* | 1.5-3% | 1 > 2 & 4  3 > 2,4 & 5  6 > 2,4 & 5 |
|  | 037 | *Oscillibacter* | 2-5% | 2 > 1,4,5 & 6  3 > 1,4 & 5 |
|  | 278 | *Coprococcus* | 5-6% | 1 > All |
| Week 14 | 002 | *Meniscus* | 10-20% | 1 > 2,3,4 & 6  5 > 2,3,4 & 6 |
|  | 003 | *Meniscus* | 10-25% | 1 > 2  5 > 2  6 > 2,3 & 4 |
|  | 004 | *Prevotella* | 2-8% | 2 > All  3 > 4 & 6 |
|  | 016 | *Parasporobacterium* | 2-3% | 6 > All |
|  | 019 | *Meniscus* | 2-5% | 1 > 2  3 > 2  5 > 4  6 > 1,2,3 & 4 |
|  | 045 | *Meniscus* | 0.5-1.5% | 1 > 3,4 & 6  2 > 3,4,5 & 6 |
|  | 047 | *Tannerella* | 0.5%  1.5-2.5% | 2 > 1,3,4,5 & 6  3 > 1,4,5 & 6 |
|  | 155 | *Butyrivibrio* | 1.4% | 6 > 1,2,3,4 & 5 |

Footnote ^a^ The range describes how large the change was between the samples.

^b^ In this example cage 1 is enriched when compared to all other cages in OTU001
