# Supplementary material for: Age and Microenvironment Outweigh Genetic Influence on the Zucker Rat Microbiome
Source: PLoS One. 2014 Sep 18;9(9):e100916. doi: 10.1371/journal.pone.0100916 (PMC4169429; doi:10.1371/journal.pone.0100916)
Supplement: Table S5 — Significant differences in the relative abundances of Bacteroidetes and Firmicutes between cages (no other phyla were found to be significantly different). Level of significance: * P<0.05; ** P<0.01; *** P<0.001. Difference between means of cages assessed using one-way ANOVA, followed by Tukey-Kramer multiple comparisons test. (DOCX) [file pone.0100916.s021.docx]

Table S5: Significant differences in the relative abundances of *Bacteroidetes* and *Firmicutes* between cages (no other phyla were found to be significantly different).

|  | ***Bacteroidetes*** | | | | ***Firmicutes*** | | | |
| --- | --- | --- | --- | --- | --- | --- | --- | --- |
| **Cage comparison:** | **Week 5** | **Week 7** | **Week 10** | **Week 14** | **Week 5** | **Week 7** | **Week 10** | **Week 14** |
| Cage 1 vs. Cage 2 |  |  |  |  |  |  |  |  |
| Cage 1 vs. Cage 3 | *** | * |  |  | *** | * |  |  |
| Cage 1 vs. Cage 4 |  | ** |  | ** | * | ** |  | * |
| Cage 1 vs. Cage 5 |  | *** |  |  |  | *** |  |  |
| Cage 1 vs. Cage 6 |  | *** |  |  |  | *** |  |  |
| Cage 2 vs. Cage 3 | *** |  |  |  | *** |  |  |  |
| Cage 2 vs. Cage 4 | * | ** |  | * | * | ** |  | * |
| Cage 2 vs. Cage 5 |  | *** |  |  |  | *** |  |  |
| Cage 2 vs. Cage 6 |  | *** |  |  |  | *** |  |  |
| Cage 3 vs. Cage 4 |  |  |  | * |  |  |  | * |
| Cage 3 vs. Cage 5 | ** |  |  |  | ** |  |  |  |
| Cage 3 vs. Cage 6 | *** | * |  |  | *** | * |  |  |
| Cage 4 vs. Cage 5 |  |  |  | ** |  |  |  | ** |
| Cage 4 vs. Cage 6 | * |  |  | * |  |  |  | * |
| Cage 5 vs. Cage 6 |  |  |  |  |  |  |  |  |

**Level of significance: * P < 0.05; ** P < 0.01; *** P < 0.001. Difference between means of cages assessed using one-way ANOVA, followed by Tukey-Kramer multiple comparisons test.**
