# Supplementary material for: Age and Microenvironment Outweigh Genetic Influence on the Zucker Rat Microbiome
Source: PLoS One. 2014 Sep 18;9(9):e100916. doi: 10.1371/journal.pone.0100916 (PMC4169429; doi:10.1371/journal.pone.0100916)
Supplement: Table S6 — Significant differences in the relative abundances of families between cages (no other families were found to be significantly different). Level of significance: * P<0.05; ** P<0.01; *** P<0.001. Difference between means of cages assessed using one-way ANOVA, followed by Tukey-Kramer multiple comparisons test. Univariate statistical comparison of all cages was not possible at week 10 due to the small sample numbers per cage (n<3). (DOCX) [file pone.0100916.s022.docx]

**Table S6:** Significant differences in the relative abundances of families between cages (no other families were found to be significantly different).

_
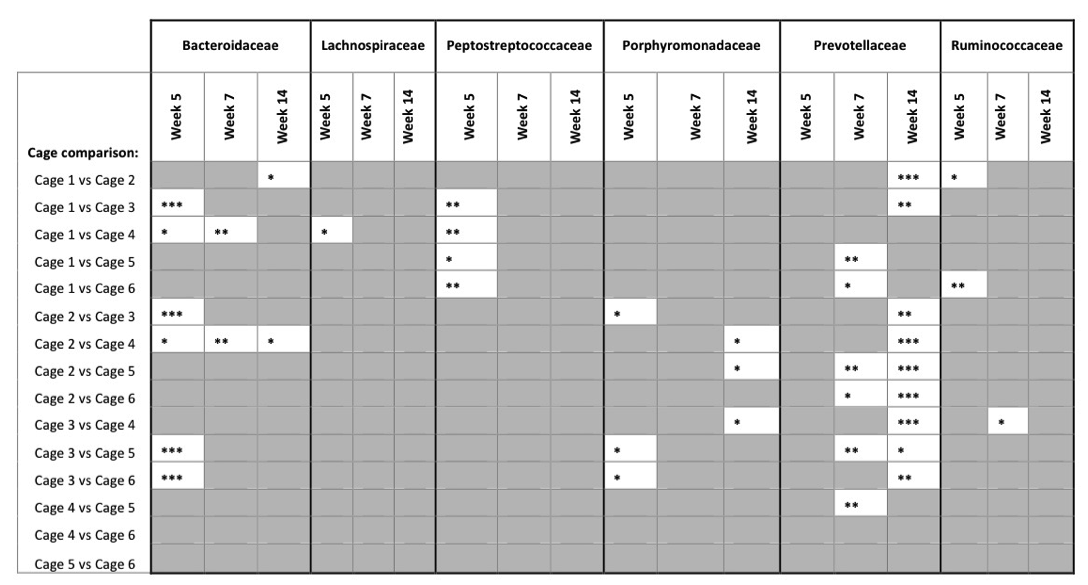
_

**Level of significance: * P < 0.05; ** P < 0.01; *** P < 0.001. Difference between means of cages assessed using one-way ANOVA, followed by Tukey-Kramer multiple comparisons test. Univariate statistical comparison of all cages was not possible at week 10 due to the small sample numbers per cage (n < 3).**
